# Characterization of macroalgal-associated microbial communities from shallow to mesophotic depths at Manawai, Papahānaumokuākea Marine National Monument, Hawai‘i

**DOI:** 10.7717/peerj.16114

**Published:** 2023-10-03

**Authors:** Gabrielle M. Kuba, Heather L. Spalding, Kristina M. Hill-Spanik, Taylor M. Williams, Monica O. Paiano, Alison R. Sherwood, Brian B. Hauk, Randall K. Kosaki, Heather Fullerton

**Affiliations:** 1 Department of Biology, College of Charleston, Charleston, SC, United States; 2 Department of Biology, Grice Marine Laboratory, College of Charleston, Charleston, SC, United States; 3 School of Life Sciences, University of Hawai‘i at Mānoa, Honolulu, HI, United States; 4 Cooperative Institute for Marine and Atmospheric Research, University of Hawai‘i at Mānoa, Honolulu, HI, United States; 5 Papahānaumokuākea Marine National Monument, National Oceanic and Atmospheric Administration, Honolulu, HI, United States; 6 Center for the Exploration of Coral Reef Ecosystems (XCoRE), Bishop Museum, Honolulu, HI, United States

**Keywords:** Macroalgae, Microbiomes, Marine, Invasive algae, Native algae

## Abstract

The Papahānaumokuākea Marine National Monument, Hawai‘i, is one of the most isolated and protected archipelagos in the world, making it a natural laboratory to examine macroalgal-microbial diversity because of limited direct anthropogenic impacts. We collected the most abundant macroalgae from nine sites ranging from shallow subtidal (1.5 m) to mesophotic (75 m) depths around Manawai (Pearl and Hermes Atoll). We characterized the macroalgal bacterial communities *via* high-throughput amplicon sequencing and compared the influence of host phylum, species, site, and depth on these relationships at a single atoll. Ochrophyta species had the lowest bacterial diversity compared to Chlorophyta and Rhodophyta. Site and/or depth may influence the microbial community structure associated with *Microdictyon setchellianum*, indicating a possible disconnect of these microbial communities among habitats. *Chondria tumulosa*, a cryptogenic species with invasive traits, differed in associated microbiota compared to the native *Laurencia galtsoffii*, an alga from the same family collected at the same site and depth. While there was overlap of bacterial communities across sites for some algal species, the majority had minimal macroalgal-microbial community connectivity across Manawai. This mesophotic system, therefore, did not appear to be refugia for shallow water coral reefs at microscopic scales. Additional studies are required to identify other significant influences on microbial community variation.

## Introduction

Marine macroalgae are siphonous or multicellular photosynthetic eukaryotes that serve as major ecosystem engineers and foundational species from shallow to mesophotic depths (Schiel & Foster, 2006; Spalding et al., 2019). Macroalgae are classified into three phyla: Chlorophyta, Rhodophyta, and Ochrophyta (commonly known as green, red, and brown algae, respectively) and range in evolutionary, chemical, and morphological characteristics. Macroalgal species increase the biodiversity in marine ecosystems by serving as a food source for herbivores (Buschmann, 1990), providing nursery grounds and habitat for invertebrates (Haywood, Vance & Loneragan, 1995; Busetti, Maggs & Gilmore, 2017), and serving as essential settlement structures for epibionts (Fraschetti et al., 2006).

Species-specific or generalist endophytic and epiphytic bacteria can form essential relationships with their macroalgal hosts and are integral to host health and function (Egan, Thomas & Kjelleberg, 2008; Wahl et al., 2012; Singh & Reddy, 2016; Kuba et al., 2021). Previous studies have shown that distinct microbial communities associate with specific macroalgal host species and phyla (Morrissey et al., 2019; Kuba et al., 2021) and that these bacterial communities differ from those within ambient water or associated with other abiotic substrata (Dobretsov, Dahms & Harder, 2006; Burke et al., 2011b; Kuba et al., 2021). Additionally, macroalgal-associated microbial communities are variable and influenced by processes including environmental conditions (Stratil et al., 2013; Saha et al., 2020; Bonthond et al., 2020), secondary metabolite production (Hay, 1986; Armstrong et al., 2001; Dobretsov, Dahms & Harder, 2006; Goecke et al., 2010; Persson et al., 2011), and the functional composition of associated microbiota (Burke et al., 2011a; Singh & Reddy, 2014; Bonthond et al., 2021).

The Northwestern Hawaiian Islands (NWHI) consist of over 130 islands, atolls, shoals, pinnacles, seamounts, and reefs (Abbott & McDermid, 2002) and are located within the Papahānaumokuākea Marine National Monument (PMNM)—the world’s second-largest marine protected area and largest in the USA. The PMNM is an intact ecosystem with few local anthropogenic disturbances (*i.e*., limited nutrient loading from runoff, overfishing, and disturbance from boat traffic; Friedlander et al., 2005). The largest atoll in PMNM is Manawai (Pearl and Hermes Atoll) with 1,166 km^2^ of reef area (Page-Albins et al., 2012). Similar to other reefs within the PMNM, Manawai is characterized by a high abundance of *Microdictyon setchellianum* M. Howe, *Halimeda* J.V. Lamouroux beds, and dense crustose coralline red algal communities (Friedlander et al., 2005; Vroom et al., 2005; Page et al., 2006) from shallow to mesophotic depths (30 to 150 m; Hinderstein et al., 2010). In the Hawaiian Archipelago, Mesophotic Coral Ecosystems (MCEs) are often macroalgal dominated habitats, with macroalgal bottom cover reaching over 70% (Parrish & Boland, 2004; Spalding et al., 2019). Fleshy algal-dominated reef systems harbor higher microbial abundances compared to coral-dominated or crustose algae reef systems (Haas et al., 2016). *Microdictyon setchellianum* beds in these habitats support herbivorous fish populations and heterotrophic benthic communities (Parrish & Boland, 2004), whereas *Halimeda* algal species aid in structuring the reef by producing calcareous sediment (Harney & Fletcher, 2003) and providing habitat for cryptic fish (Langston & Spalding, 2017) and invertebrates (Fukunaga, 2008).

In 2016, a new cryptogenic red alga, *Chondria tumulosa* A.R. Sherwood & J.M. Huisman, was collected at Manawai (Sherwood et al., 2020). This species was discovered proliferating into dense mats attached to hard substrata and smothering foundation coral and native macroalgal species at Manawai (Sherwood et al., 2020), thus threatening the overall biodiversity on this reef. Understanding microbiota associated with invasive species is crucial as they may play a role in the invasion process, supporting the invasive species’ ability to adapt in newly established habitats (Aires et al., 2013).

A NOAA research cruise to Manawai in August 2019 provided a unique opportunity to intensively sample the most abundant macroalgae from shallow to mesophotic depths and to examine macroalgal microbial communities across broad spatial scales. We characterized the microbial communities using high-throughput amplicon DNA sequencing, assessed the macroalgal-associated bacterial communities by macroalgal phyla, examined the influence of depth on the community structure for one species (*M. setchellianum*) collected at six sites, examined the microbial community structure of two species in the same genus (*Halimeda* spp.) at a single site, and compared the bacterial communities found associated with a cryptogenic alga (*C. tumulosa*) and a native alga from the same family.

## Materials and Methods

### Sampling site and abiotic factors

**Figure 1:**
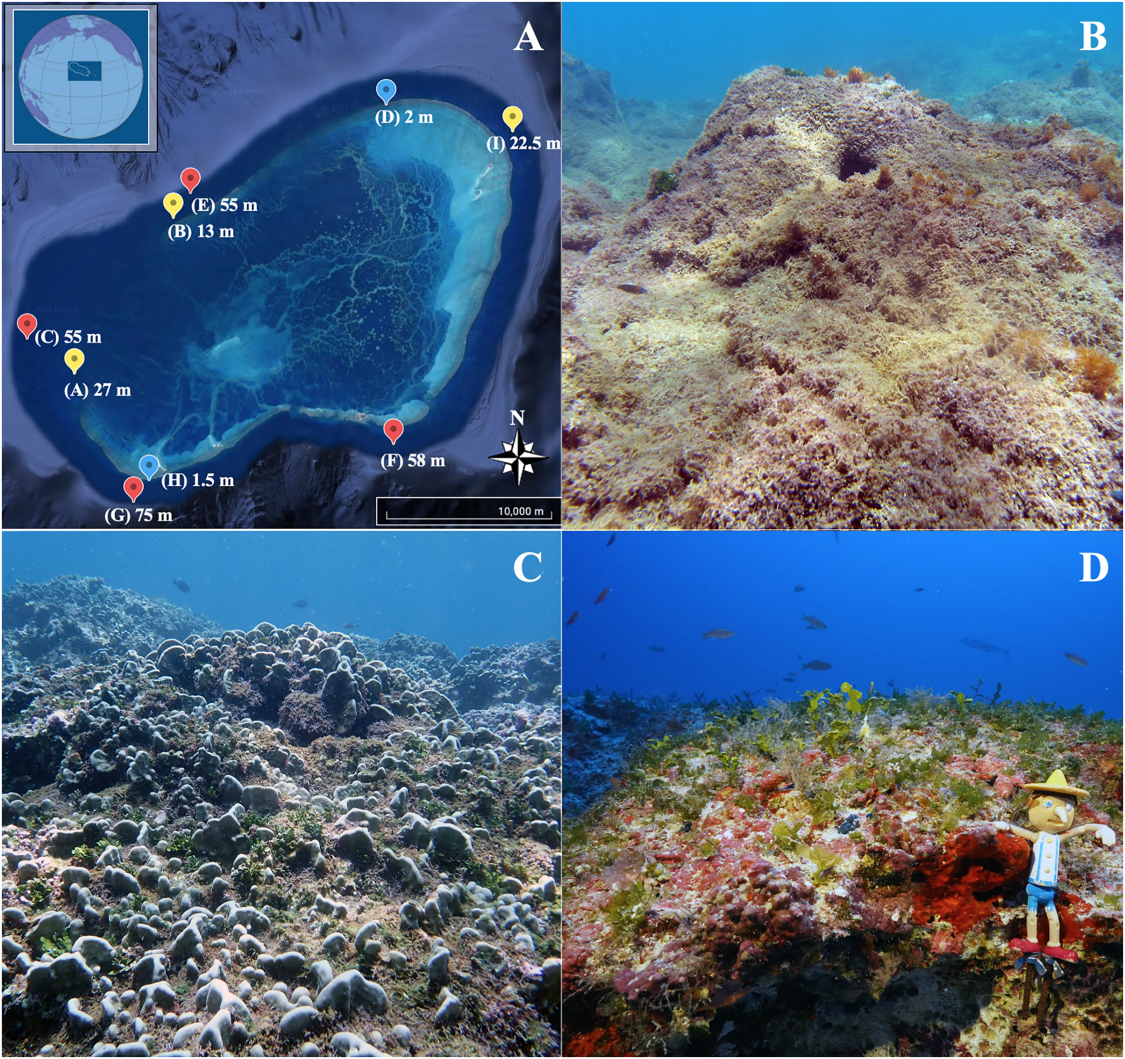
Map of the Hawaiian Archipelago and sampling sites. (A) Satellite image of Manawai (Pearl and Hermes Atoll), Northwestern Hawaiian Islands, Hawai‘i, USA (Google Earth^©^, with inset courtesy of Papahānaumokuākea Marine National Monument). Points refer to collection sites with the associated depth for the site. The color of each point indicates depth zone at the site (blue: shallow subtidal (1.5, 2 m); yellow: subtidal (13, 22.5 m); red: upper mesophotic (55, 58, 75 m)). Photographs of each zone are provided: (B) shallow subtidal, (C) subtidal, and (D) upper mesophotic, Pinocchio included for size and color reference (11.4 cm).

Samples were collected from August 03–08, 2019 from nine sites around Manawai PMNM, NWHI (Fig. 1). Field collections were approved by the Papahānaumokuākea Marine National Monument, permit number PMNM-2018-029. Collection numbers varied per site ranging from 6 to 12 samples (*n* = 6 at sites A and G; *n* = 8 at site H; *n* = 9 at sites B through F; *n* = 12 at site I). All sites varied by depth, except sites C and E. We categorized sampling into three depth zones according to Hinderstein et al. (2010): shallow subtidal (1.5, 2.0 m), subtidal (13, 22.5, and 27 m), and upper mesophotic (55, 58, and 75 m).

Light attenuation and temperature data for Manawai were characterized for each depth. Temperature data were collected using a Shearwater Petrel (Richmond, BC, Canada) wrist-mounted dive computer (upper and lower mesophotic) and Aqualung i200 (Vista, CA, USA) wrist-mounted diver computer (shallow subtidal and subtidal), which were calibrated prior to sample collection. Several minutes of immersion at the target depths were allowed for the dive computers to equilibrate with ambient temperature.

Six irradiance profiles were collected from the forereef and backreef at Manawai from July 15–20, 2021. The irradiance profiles were calculated as previously described (Spalding, Foster & Heine, 2003). Underwater irradiance was measured by lowering a spherical (4π) quantum sensor (Underwater LI-193SA; LI-COR, Lincoln, NE, USA) through the water column and storing the data in a datalogger (model 1400; LI-COR, Lincoln, NE, USA). The sensor was mounted on a lowering frame (LI-COR, Lincoln, NE, USA) and attached to the datalogger with a 20-m cable marked at 0.5-m intervals. PAR (μmol photons·m^−2^·s^−1^) was recorded every 0.5 to 1 m to a maximum depth of 15 m. Profiles were completed from the sunny side of the boat on clear days between 1100 and 1300 h. *K*_0_ was calculated from irradiance profiles according to Beer’s Law. The %SI (surface irradiance) was calculated from irradiance extrapolated from *K*_0_ at 0.01 m.

### Sample collection

Approximately one to three cm of new growth (*i.e*., the apical ends) of each most abundant macroalgal species (*n* = 3 per morphospecies) per site was collected. The most abundant macroalgal species were visually determined by experienced divers and phycologists with expertise on Hawaiian macroalgae. Each individual sample was collected into individual Whirlpak^©^ bags by the scientific dive team and sealed after collection underwater to limit any possible contamination among samples. Each sample was rinsed with 3.5% sterile artificial seawater to remove loosely attached epibionts and sand as previously described (Kuba et al., 2021). Individual thalli were then placed in RNAlater and stored overnight at 4 °C before freezing at −20 °C on the NOAA ship *Rainier*. A background seawater sample (*n* = 1) for each site was collected by filtering 50 mL of ambient seawater from each site (Boström et al., 2004) with an additional 50 mL artificial seawater control (*n* = 1) through a sterile 0.2 μm filter and preserved in 5 mL of RNAlater. This artificial seawater control was the same water used to rinse the samples prior to placing in RNAlater and served as a control for decontamination processing after assigning taxonomy. Each individual water sample was also collected into individual containers prior to filtering. The background seawater samples were collected approximately 1 m above the bottom at each site, and care was taken to collect water up current with no mixing with the benthos and represents a seawater control. These water controls serve as background controls to better understand the macroalgal-specific associations at our sample locations. This volume of seawater has an associated average DNA extraction efficiency of 92% for marine planktobacteria (Boström et al., 2004). Filters were stored at 4 °C before freezing at −20 °C. All samples for DNA analysis were shipped overnight on Techni Ice™ frozen at −80 °C from Honolulu, HI to Charleston, SC and subsequently frozen at −80 °C.

### Identification of macroalgae

**Figure 2:**
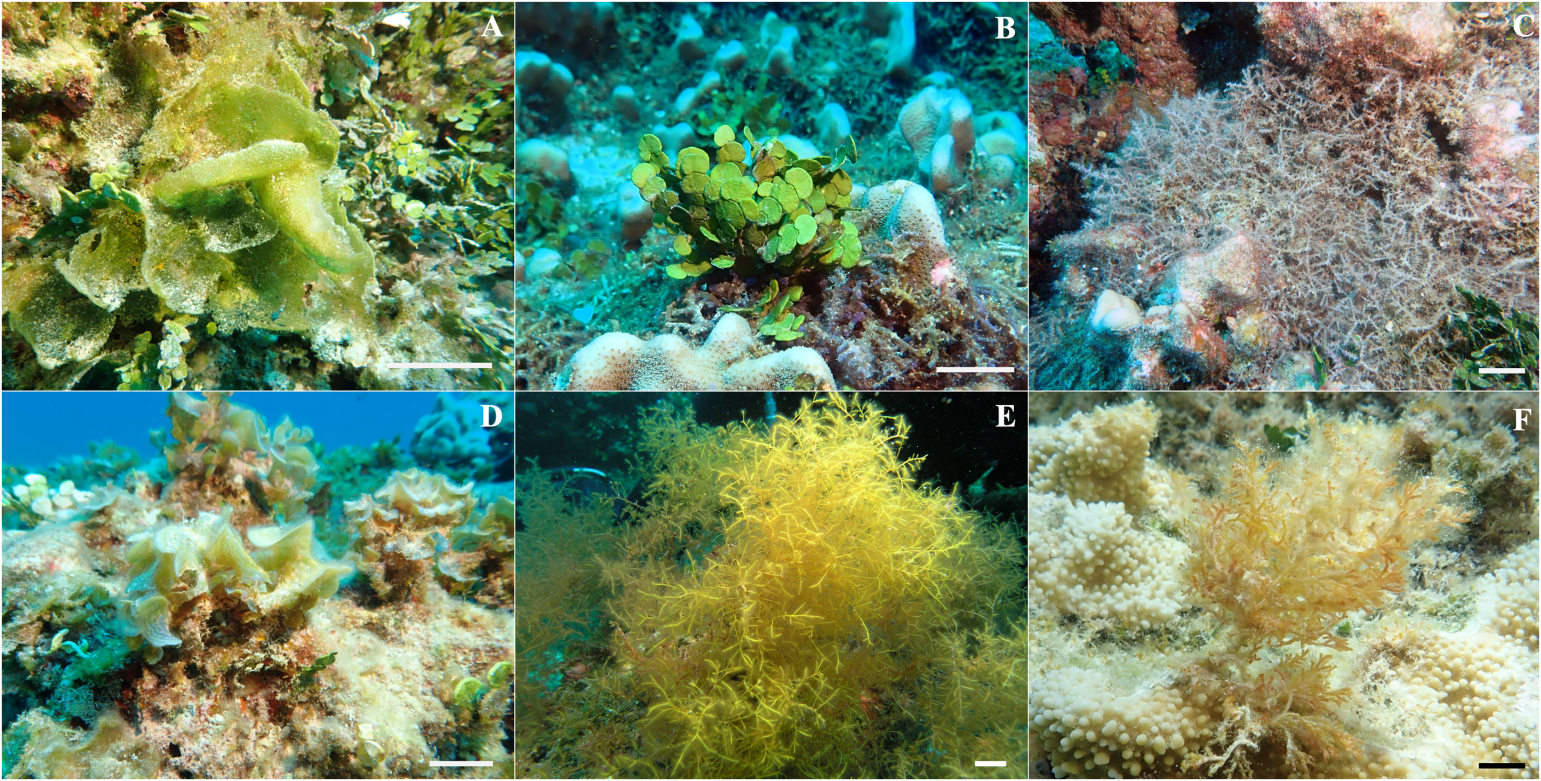
Representative *in situ* photos of macroalgal species identified at Manawai, Northwestern Hawaiian Islands used for this study. (A) *Microdictyon setchellianum* (B) *Halimeda discoidea* (C) *Chondria tumulosa* (D) *Padina* sp. (E) *Wrightiella* sp., and (F) *Laurencia galtsoffii*. Scale bar is 5 cm.

Macroalgae were identified visually based on morphological descriptions using Abbott (1999), Abbott & Huisman (2004), and Huisman, Abbott & Smith (2007) or by molecular analysis (Fig. 2; Table 1). Molecular analyses were completed and compared to voucher identifications (Table S1). For macroalgae that could not be conclusively identified by morphology, genomic DNA was extracted using an OMEGA E.Z.N.A.^®^ Plant DNA DS Kit (OMEGA Biotek, Norcross, GA, USA) or a NucleoSpin Plant II Kit (Macherey-Nagel, Düren, Germany) following the manufacturer’s protocol. For identification of the Chlorophyta specimens (vouchers NWHI 878, NWHI 1071 and NWHI 1049), a portion of the *tuf*A gene (elongation factor Tu) was amplified using primers *tuf*A_alg_up and *tuf*A_alg_do (Handeler et al., 2010). For the Rhodophyta (NWHI 1073, NWHI 1032 and NWHI 813), a portion from the DNA barcode region near the 5′ end of the mitochondrial *COI* (cytochrome *c* oxidase subunit 1) gene was generated using primers GazF1 and GazR1 (Saunders, 2005). For further identification of the Rhodophyta (NWHI 1073, NWHI 1032 and NWHI 813), the *rbcL* (ribulose-1, 5-bisphosphate carboxylase/oxygenase large subunit) gene was amplified as two overlapping fragments using the primer pairs rbcLF7 and R898 (Gavio & Fredericq, 2002; Kim, Kim & Nelson, 2010) and rbcLF762 and R1381 (Kim, Kim & Nelson, 2010). Amplification conditions consisted of 94 °C for 3 min followed by 35 cycles of 30 s at 94 °C, 30 s annealing at 55 °C and 5 min synthesis at 72 °C, followed by a final extension at 72 °C for 7 min for *tufA*, and 4 min at 96 °C for denaturation, followed by 35 cycles of 60 s at 94 °C, 60 s at 42 °C and 90 s at 72 °C, with a final 10 min extension cycle at 72 °C and soak cycle at 10 °C for *rbcL*. The barcode region was amplified as previously described (Saunders, 2005). Successful PCR products were cleaned with ExoSAP-IT™ Express PCR Product Cleanup kit and submitted for sequencing at GENEWIZ (South Plainfield, NJ, USA). Raw sequence reads for each gene were assembled, edited, and aligned using the MUSCLE v. 3.8.425 plug-in (Edgar, 2004) in Geneious Prime 2021.0.3 (Fukunaga, 2008). Molecular sequences generated for the six macroalgae species were compared to those in GenBank using BLAST (Basic Local Alignment Tool; www.ncbi.nlm.nih.gov).

**Table 1:** Macroalgal specimen descriptions and their associated characteristics.

Phylum	Species identification	Sampling site*	Depth (m)	Thallus complexity	Calcification
Chlorophyta	*Halimeda discoidea*	A, H, I	1.5, 22.5, 27	Flattened segments	Calcified
	*Halimeda velasquezii*	B, D, I	2, 13, 22.5	Flattened segments	Calcified
	*Microdictyon setchellianum*	A, B, C, F, H, I	1.5, 13, 22.5, 27, 55, 58	Coarse mesh	Uncalcified
	*Neomeris annulata*	H	1.5	Club-shaped	Calcified
	*Umbraulva kaloakulau*	G	75	Distromatic blade	Uncalcified
Ochrophyta	*Dictyota ceylanica*	E	55	Flattened dichotomous branches	Uncalcified
	*Distromium* sp.	E	55	Fan-shaped	Uncalcified
	*Padina moffitiana*	E	55, 58	Fan-shaped	Lightly Calcified
	*Padina* sp.	F	58	Fan-shaped	Lightly Calcified
	*Sporochnus dotyi*	F	58	Alternately branched	Uncalcified
Rhodophyta	*Chondria tumulosa*	B, D	2, 13	Terete branches	Uncalcified
	*Dudresnaya babbittiana*	C	55	Cylindrical irregularly branches	Uncalcified
	*Galaxaura filamentosa*	C	55	Flattened dichotomous branches	Calcified
	*Gracilaria* sp.	G	75	Unbranched	Uncalcified
	*Laurencia galtsoffii*	D	2	Erect distichous branches	Uncalcified
	*Wrightiella* sp.	I	22.5	Erect distichous branches	Uncalcified
Background seawater	Seawater control	All	All	NA	NA

**Notes:**

Characteristics include macroalgal phylum, collection depths (m), thallus complexity, calcification level, and status as cryptogenic or native. *N* = 3 per species and site except for *Neomeris annulata* where *n* = 2. Background seawater samples were also collected at each of the nine sampling sites (*n* = 1 per site). All macroalgal species were native to Manawai, except *Chondria tumulosa*, which has been described as a cryptogenic species.

* Refer to Fig. 1.

### DNA extraction, library preparation, sequencing

All samples were thawed on ice and DNA was extracted from individual thalli using the FastDNA SPIN Kit (MP Biomedicals, Santa Ana, CA, USA) following the manufacturer’s protocol with minor modifications. Approximately 0.5 g of each algal sample was weighed into the lysis matrix E tube using ethanol flame-sterilized forceps. Thalli were split into two lysis tubes if an individual was >0.5 g. Each seawater control filter was cut and divided into lysis matrix E tubes to a final weight of 0.5 g. Extractions were completed on entire algal samples to include both associated epibionts and endobionts. Lysis tubes were placed in a cold aluminum rack and homogenized at 3,800 RPM for 30 s (BioSpec BeadBeater, Bartlesville, OK, USA). Bead beating was repeated twice with an incubation period of 30 s on ice in between each homogenization. DNA was eluted twice with 50 µl 0.1 mM Tris (pH 8.0). If thalli were split, eluted DNA was combined into one tube before further processing. DNA was quantified with a Qubit 3.0 fluorometer using the dsDNA high sensitivity kit (ThermoFisher Scientific, Waltham, MA, USA).

PCR was performed using the protocol from Klindworth et al. (2013) targeting variable regions 3 and 4 of the bacterial SSU rRNA gene as previously described (Kuba et al., 2021). Negative controls were included through PCR protocols, however no blank extractions were included. Primers possessed Illumina overhang sequences that were used for ligation of index sequences for all macroalgal and seawater controls. Cleaned amplicons were indexed and sequenced on an Illumina MiSeq per the manufacturer’s protocol, generating 2X300 base pair (bp) paired-end reads.

### Bioinformatics of macroalgae-associated bacterial communities

Demultiplexed sequences with adapters removed were analyzed as previously described (Kuba et al., 2021). Amplicon sequence variants (ASVs) were inferred per MiSeq run (Callahan et al., 2016). Chimera identification and removal was performed after runs were merged using the “removeBimeraDenovo” command as implemented in dada2 (version 1.18). Each biological triplicate was kept as an independent sample for further processing to compare variation among individuals.

Statistical analyses were performed using R version 4.0.1 (R Core Team, 2010) and visualizations were generated using ggplot2 (Wickham, 2016), phyloseq (McMurdie & Holmes, 2013), and microbiome (Lahti & Shetty, 2017). Differential abundance analyses were utilized using the DESeq2 tool (Love, Huber & Anders, 2014) with an alpha <0.01 to identify the amplicon sequence variants (ASVs) contributing to overall differences among samples. This tool accounts for low dispersion estimates and is consistent across studies with varying replicates (Love, Huber & Anders, 2014). Data were then normalized using variance stabilizing transformation implemented with DESeq2 to compare microbiota across samples. Rarefaction of the number of reads per sample were visualized using the “rarecurve” command in vegan version 2.0–4 (Oksanen et al., 2016). Read abundances were transformed prior to calculating Euclidean distances. Visualizations of hierarchical clustering were performed with variance stabilized Euclidean distances. This transformation was applied due to the low associated false positive rate (McMurdie & Holmes, 2014). Alpha diversity was estimated using multiple indices (Table S3), but we chose to report the Simpson’s diversity index because of its emphasis on species evenness rather than richness as compared to the Shannon-Weaver index and Faith’s phylogenetic diversity; Simpson’s diversity index values closer to zero have lower microbial diversity. An ANOVA was run on each individual diversity model and a *post-hoc* Kruskal-Wallis test was ran on each individual model to compare between algal phylum/seawater. Beta diversity was visualized using a non-multidimensional scaling (NMDS) plot based on the Bray-Curtis dissimilarity metric. Bray-Curtis dissimilarity was applied rather than VST-stabilized Euclidean distances to account for distance and microbial species identity. Statistical tests, ANOVA, PERMANOVA, PERMDISP2, and ANOSIM were performed in vegan (Oksanen et al., 2016). If groups were non-homogenous, an ANOSIM was performed where the R-value ranges from 0 to 1 representative of indistinguishable to well-separated communities, respectively (Clarke & Gorley, 2001). Taxonomic distributions of macroalgal-associated microbiota were visualized using a heat map through ggplot2 (Wickham, 2016) with the associated cutoff for ASV relative abundance set to >0.05% and presence in >3 samples.

### Data availability

Sequence data are available through NCBI Sequence Read Archive (SRA) BioProject number PRJNA833318. Macroalgal sequences used for taxonomic identification are available with accession numbers OR066430–OR066437, OK448437, OK448460 (Table S1). Relevant code used for the bioinformatics work is available on GitHub (https://doi.org/10.5281/zenodo.7975190).

## Results

### Sample collection and abiotic factors

The most abundant macroalgae at Manawai were collected at each site (Fig. 1; Table 1). Key characteristics of these species are noted in Table 1. The calcification levels described include uncalcified, lightly calcified, and calcified. The shallow subtidal (1.5 to 2 m) temperatures ranged from 26–27 °C, subtidal (13 to 27 m) temperatures from 25–27 °C, and upper mesophotic (55 to 75 m) from 22–27 °C. The average diffuse attenuation coefficient (K_o_) was −0.118 m^−1^ (±0.01 m^−1^ SE). Shallow subtidal irradiance ranged from 1,003 to 1,064 μmol photons m^−2^ s^−1^, with 79% to 84% surface irradiance (SI). Subtidal irradiance ranged from 53 to 274 μmol photons m^−2^ s^−1^, with 4% to 22% SI. Lastly, the upper mesophotic was characterized by irradiance ranging from 0.18 to 1.93 μmol photons m^−2^ s^−1^ and 0.014% to 0.152% of SI. The average 10% optical depth was 19 m (2.3/K_o_), and the 1% optical depth was 39 m (4.6/K_o_) (Kirk, 1994). Light attenuation and temperature data are provided in Table S2.

### Molecular identification of macroalgae

Four of the six macroalgal species that could not be identified based on morphology alone were identified to at least genus level using BLAST. The Chlorophyta macroalgal voucher NWHI 1049 was 100% identical to *Umbraulva kaloakulau* H.L. Spalding & A.R. Sherwood (Chlorophyta) sequences, while the other two Chlorophyta algal vouchers were both identified as *Halimeda velasquezii* W.R. Taylor (Chlorophyta), having 99.2% and 99.6% (NWHI-878 and NWHI-1071, respectively) similarity with *H. velasquezii* sequences in GenBank, as well as additional morphological confirmation. For molecular identification of the Rhodophyta, two markers were used: *COI* and *rbcL*, and combined with morphological analysis. BLAST results for voucher NWHI-813 indicated it was *Laurencia galtsoffii* M. Howe with 99.8% of similarity for the *COI* marker (amplification of *rbcL* was not successful for this specimen). *COI* sequence from voucher NWHI-1032 yielded 93.2% similarity with *Gracilaria shimodensis* (Rhodophyta) and was 96.3% to *G. hayi*, while *COI* sequence from voucher NWHI-1073 was only 85.8% similar to *Symphyocladiella gracilis* (Rhodophyta) and *rbcL* sequence was 93.5% similar to *Wrightiella tumanowiczii* (Rhodophyta). Because additional morphological analysis is necessary to attribute species names to these vouchers, we referred to these specimens as *Gracilaria* sp. and *Wrightiella* sp.

### Characterization of macroalgal associated microbial communities at Manawai

A total of 77 macroalgal specimens representing 15 species, nine background seawater samples, and one artificial seawater control were sequenced, resulting in 5,357,308 total reads representing 50,445 ASVs with an average length of 415 ± 30 bp after quality control, filtering, and chimera filtering. Sequencing appeared to capture the overall diversity for each specimen based on the rarefaction curve plateau across all samples (Fig. S1). Sample read depth varied while characterizing microbial diversity (medium, 18,722; median, 165,447; maximum, 364,285 across samples).

**Figure 3:**
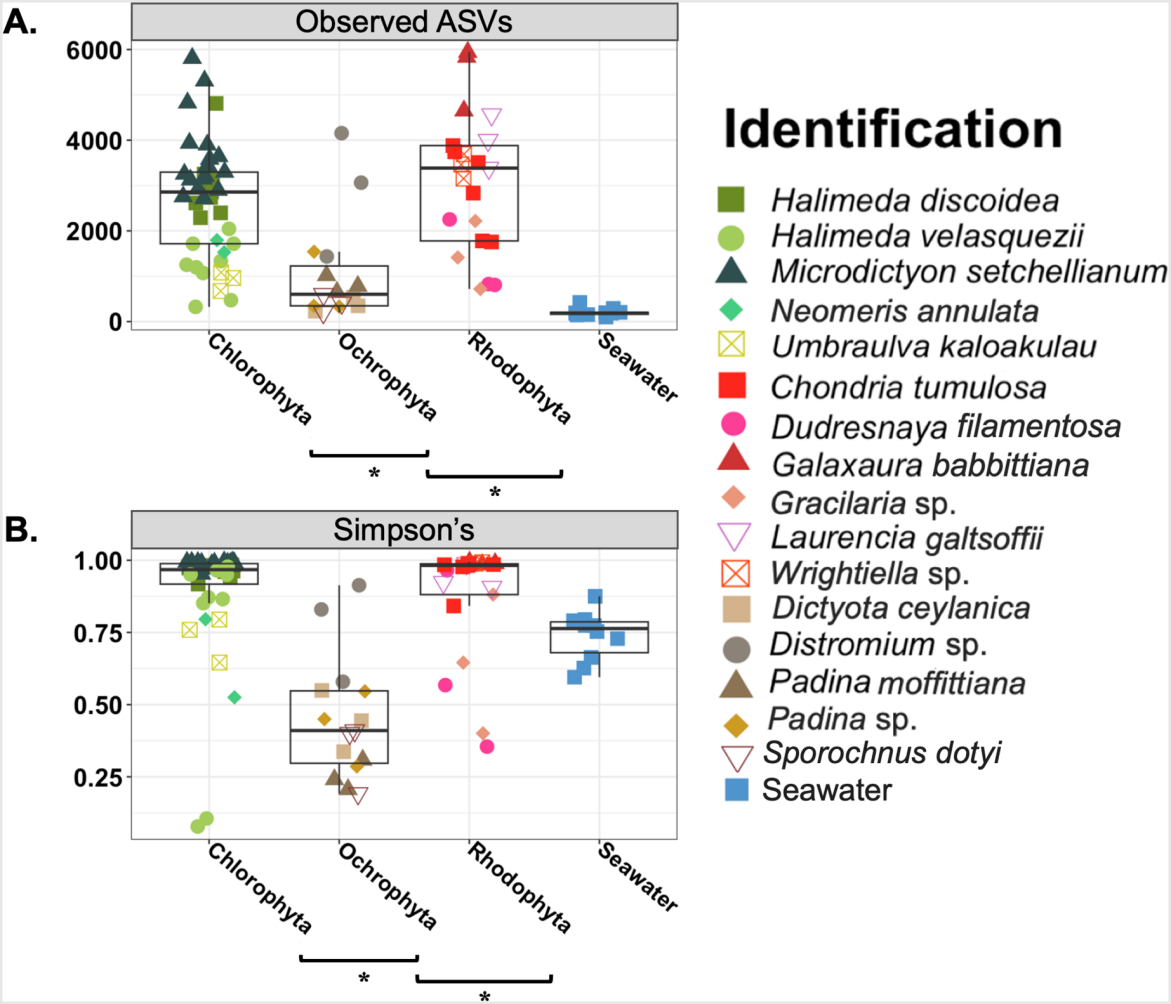
Boxplot of observed amplicon sequence variants (Observed ASVs) and Simpson’s indices. Asterisks indicate significant differences based on an Kruskal-Wallis pairwise comparison for each. ANOVA ran on Shannon (*p* = < 0.001) and PD (*p* = < 0.001) were both statistically significant.

Overall the average microbial richness was highest for the Chlorophyta samples followed by Rhodophyta and then Ochrophyta, as measured by number of observed ASVs, Shannon index, Simpson’s index, and Faith’s phylogenetic diversity (Fig. 3 and Fig. S2). The number of observed ASVs for the background seawater was lower than all macroalgal samples (Fig. 3). Microbial communities associated with macroalgae at Manawai significantly overlapped (ANOSIM R = 0.1955; *p* = 0.0039) (Fig. S3). The associated phylogenetic diversity was significantly different between Ochrophyta and both Chlorophyta and Rhodophyta (Fig. 3 and Fig. S2; Tables S3 and S4). According to the Simpson’s index, microbial diversity was lowest for Ochrophyta (Fig. 3). The total number of observed ASVs across all Ochrophyta specimens ranged from 229 (*Distromium* sp.) to 5,942 (*Dictyota ceylanica* Kützing) both from site E at 55 m (Table S3). The number of observed ASVs varied among species and among biological triplicates. A specimen of *H. velasquezii* (Chlorophyta) collected at 2 m depth had the lowest diversity based on the Simpson’s index (0.078), followed by another replicate of the same species at the same depth and site (0.106).

**Figure 4:**
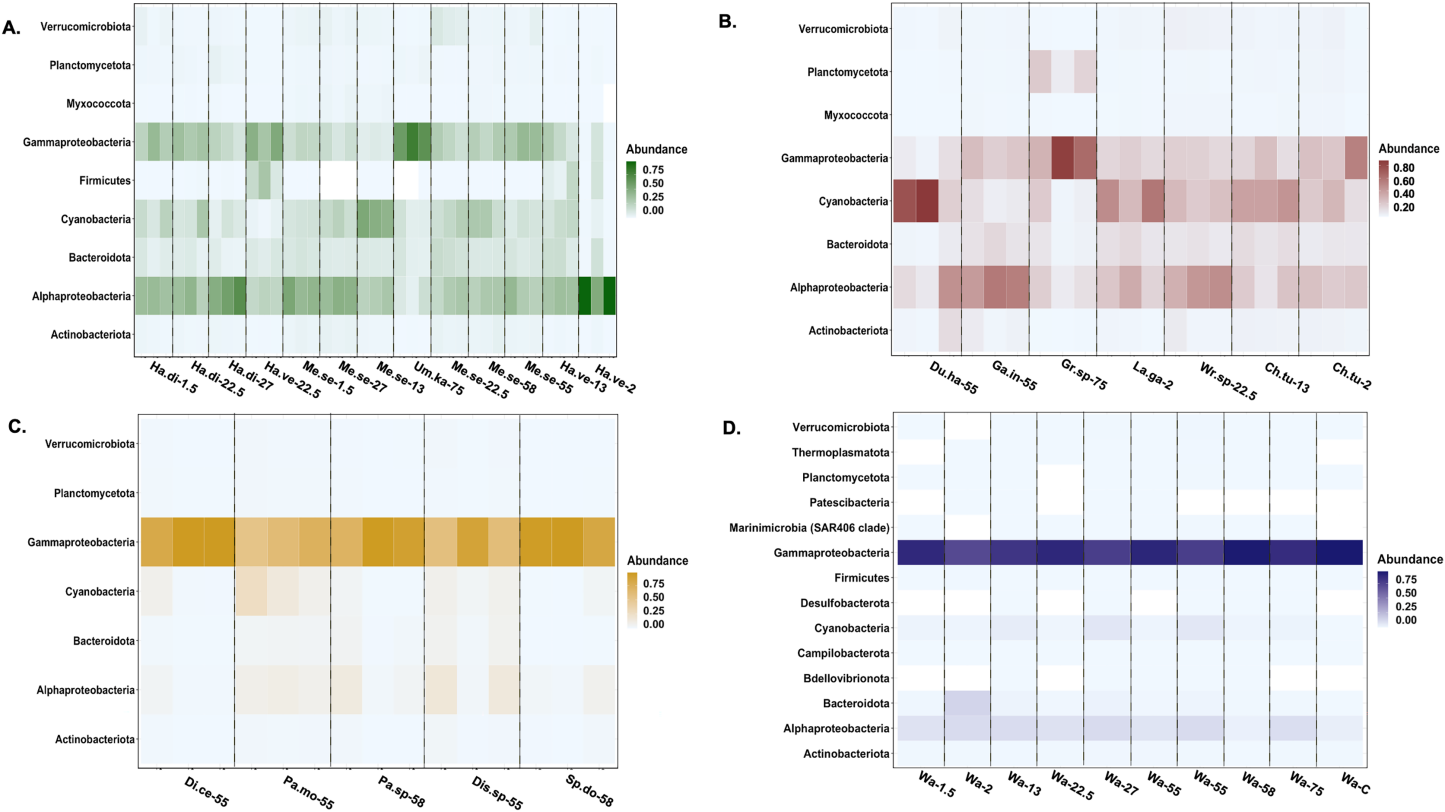
Heatmap of the taxonomic distribution of macroalgal-associated bacteria class of the top 3% in relative abundance. The relative abundance of bacteria phylum or class is provided for each sample. Each macroalgal phylum ((A) Chlorophyta, (B) Rhodophyta, (C) Ochrophyta) is considered, as well as the background seawater control (D). Each sample (*n* = 77) is shown except for seawater control samples (*n* = 10). Sample identification corresponds to the given genus and species (Ha.di, *Halimeda discoidea*; Ha.ve, *Halimeda velasquezii*; Mi.se, *Microdictyon setchellianum*; Um.ka, *Umbraulva kaloakulau*; Ch.tu; *Chondria tumulosa*; Du.ha, *Dudresnaya hawaiiensis;* Ga.in, *Galaxaura indica;* Gr.sp, *Gracilaria* sp.; La.ga, *Laurencia galtsoffii;* Wr.sp, *Wrightiella* sp.; Di.ce, *Dictyota ceylanica*; Pa.mo, *Padina moffittiana*; Pa.sp, *Padina* sp.; Sp.do, *Sporochnus dotyi*; Di.sp, *Distromium* sp.; Wa, Seawater control).

Actinobacteriota, Alphaproteobacteria, Bacteroidota, Cyanophyceae, Gammaproteobacteria, Planctomycetota, and Verrucomicrobiota were the most relatively abundant bacterial classes associated with all macroalgal phyla (Fig. 4). Myxococcota was not associated with Ochrophyta, and Firmicutes was unique to Chlorophyta. The background seawater samples varied in associated bacterial taxa, but Gammaproteobacteria sequences had the highest relative abundance across all controls (Fig. 4). Ochrophyta were associated with fewer bacterial taxa as compared to the other phyla and the seawater controls. Gammaproteobacteria associated with Ochrophyta (Pseudomonadales and Alteromonadales) showed the highest relative abundance.

**Figure 5:**
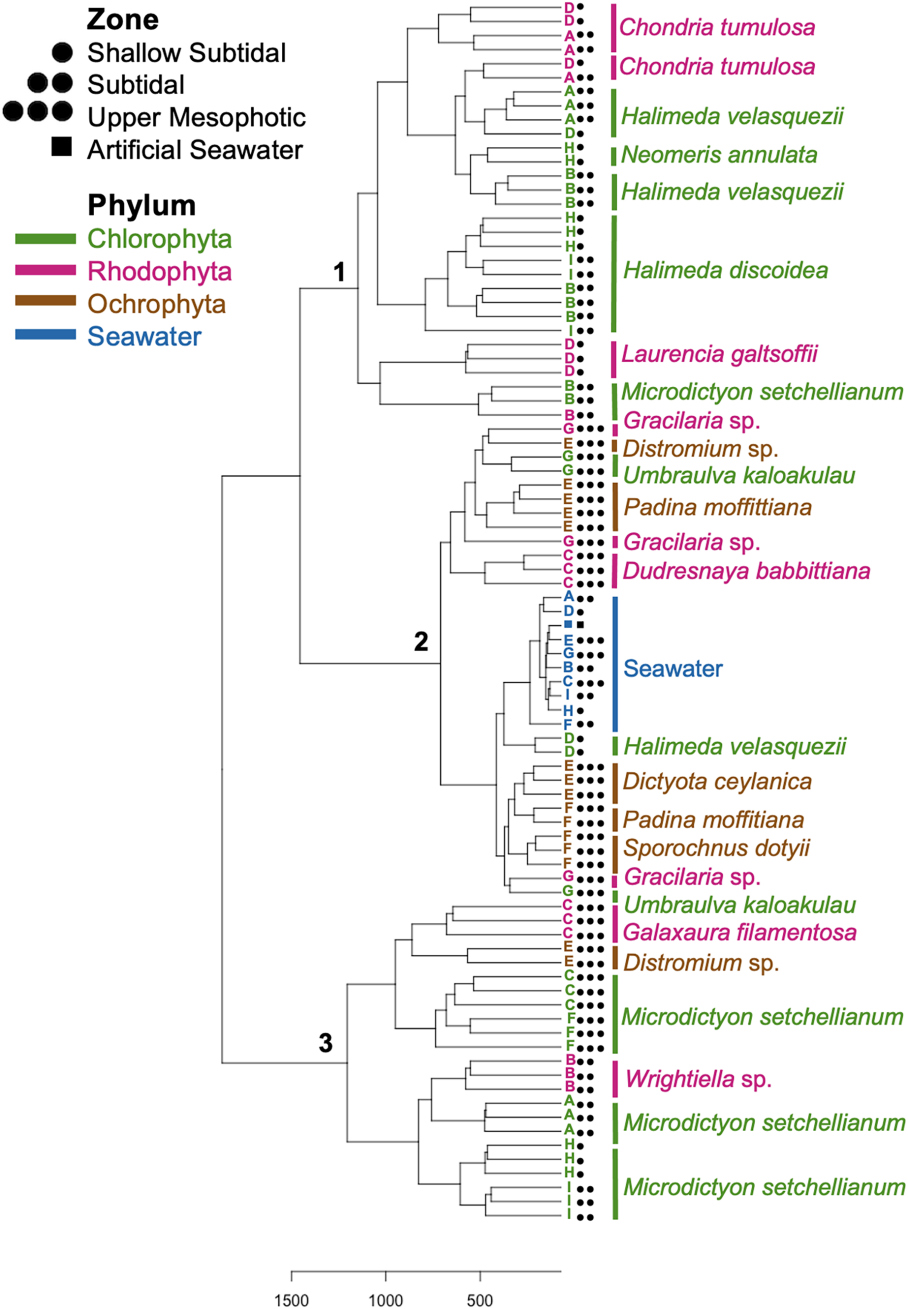
Dendrogram of hierarchical clustering based on Euclidean distances of microbial communities associated with macroalgal species at Manawai, Northwestern Hawaiian Islands, Hawai’i, USA. Distances were subjected to variance stabilizing transformation (VST). Macroalgal phylum is indicated by color: Chlorophyta (green), Ochrophyta (brown), Rhodophyta (red), seawater control (blue). The depth zone (shallow subtidal: 1.5, 2 m; subtidal: 13, 22.5 m; upper mesophotic: 55, 58, 75 m) where each specimen was collected is indicated by shape.

Hierarchical clustering based on Euclidean distances separated the microbial communities into three groups (Fig. 5). Each of these clusters contained multiple phyla and species; however, only Rhodophyta and Chlorophyta were represented in cluster one (Fig. 5). Throughout the three clusters, communities from each macroalgal species clustered together, with only a few exceptions (Fig. 5). *U. kaloakulau* from 75 m depth was found in two different clusters, with similar microbial communities to Ochrophyta and Rhodophyta species. The seawater controls grouped together in cluster two regardless of collection site (Fig. 5). Microbial community structure from the same macroalgal species was similar. All Ochrophyta species and specimens were clustered in the second cluster.

### Spatial comparison of *M. setchellianum* microbial community structure

**Figure 6:**
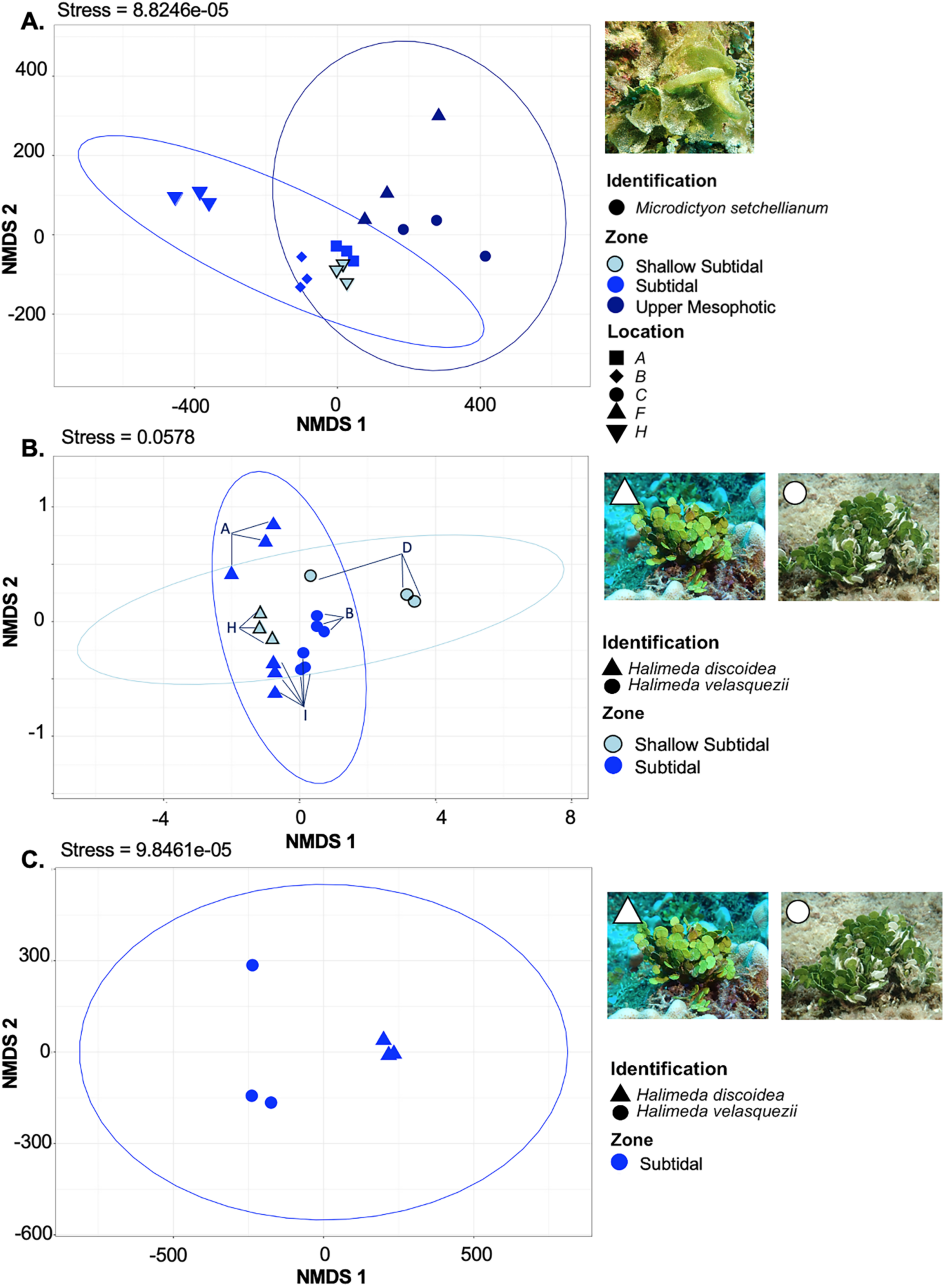
Non-metric multidimensional scaling plot generated using Bray-Curtis dissimilarities for microbial communities associated with three Chlorophyta species: *Halimeda discoidea, H. velasquezii*, and *Microdictyon setchellianum* (*n* = 3 each). (A) *M. setchellianum* from six sites and three depth zones, (B) *H. discoidea* and *H. velasquezii* from four sites and two depth zones, (C) *H. discoidea* and *H. velasquezii* from one site where they co-occurred at 22.5 m. Ellipses represent 95% confidence intervals.

*M. setchellianum* was the most abundant macroalga at Manawai, which resulted in its collection from six sites spanning all depth zones. This replication allowed for the examination of microbial communities over depths and depth zones of a single macroalgal species. Based upon overall variation across sites, samples were analyzed by depth zone rather than absolute depth. Specimens of *M. setchellianum* collected at sites A (1.5 m) and I (27 m) were associated with the highest diversity (>0.99). Most *M. setchellianum* communities were found within cluster three of the hierarchical clustering dendrogram (Fig. 5). Samples from sites C and F (*n* = 3 at both sites), both from the upper mesophotic zone, were found in a subgroup of cluster three that only contain macroalgae sampled from the upper mesophotic (Fig. 5). All seawater were found in a subgroup of cluster two rather than as an outgroup, indicating some similarity between microbiota associated with macroalgal hosts and surrounding seawater (Fig. 5). The *M. setchellianum* communities from sites A, B, and H (*n* = 3 at all sites) from the shallow subtidal and subtidal zones form the other subgroup of cluster three, along with *Wrightiella* sp. (also from the subtidal). The outliers were the subtidal *M. setchellianum* communities from site I, which were found within cluster one (Fig. 5). Microbial communities associated with *M. setchellianum* overlapped significantly across sites (ANOSIM R = 0.38; *p* = 0.0049) and based on collection depth zone (ANOSIM R = 0.36; *p* = 0.0059). Shallow subtidal *M. setchellianum* microbial communities appeared to differ the most as confidence intervals for subtidal and upper mesophotic communities overlapped (ANOSIM R = 0.11; *p* = 0.002) (Fig. 6A).

### Microbial community structure of *Halimeda* spp.

Two *Halimeda* species (*H. discoidea* Decaisne and *H. velasquezii*) were abundant at three shallow subtidal and subtidal sites and co-occurred at one site (I, 22.5 m depth). All *Halimeda* spp. bacterial communities were within cluster one except for *H. velasquezii* bacteria from site D from the shallow subtidal (Fig. 5). Microbial associations were significantly different based on site when each species was examined individually (PERMANOVA *p* = 0.003 for *H. discoidea*, *p* = 0.007 for *H. velasquezii*) (Fig. 6B). Close associations were also apparent among the biological triplicates for both *Halimeda* spp. at each site (Figs. 5, 6B). Analysis by PERMANOVA supported a difference in overall microbial communities associated with *Halimeda* spp. based on site (*p* = 0.001) (Fig. 5B). Bacterial communities associated with *H. discoidea* and *H. velasquezii* at all sites were different but overlapped (ANOSIM R = 0.56; *p* = 0.0001) (Fig. 6B). Microbial communities were well separated between these species at the single site where they co-occurred, however, this separation was not significant (ANOSIM R = 0.82, *p* = 0.1) (Fig. 6C).

### Microbial communities associated with the cryptogenic alga *C. tumulosa*

*C. tumulosa* was collected from two sites (B and D, *n* = 3 at each site). At site D (2 m), we also collected specimens of native rhodophyte *L. galtsoffii* (*n* = 3), allowing us to examine differences in microbial communities between a cryptogenic and native alga in the same family (Rhodomelaceae). Specimens of *C. tumulosa* and *L. galtsoffii* were associated with five overlapping dominant associated bacterial class, similar to other rhodophycean algae sampled (Fig. S2B). However, *C. tumulosa* was associated with two unique dominant bacterial classes: Gammaproteobacteria and Cyanobacteriia (Fig. 4B). The Simpson’s diversity index associated with all *C. tumulosa* averaged 0.959 and *L. galtsoffii* averaged 0.963 (Table S3). All bacterial communities from *C. tumulosa* specimens were in cluster one in the hierarchical clustering analysis but in separate subgroups, independent of sampling site (Fig. 5). Communities from *L. galtsoffii* were also found in cluster one, but in a different subgroup (Fig. 5). Microbial community composition from these two host species were separated without statistical significance (ANOSIM R = 1; *p* = 0.1, Fig. S4).

## Discussion

The microbial communities associated with abundant macroalgal species at Manawai were similar to communities previously identified associated with marine macroalgae (Armstrong et al., 2001; Wiencke & Bischof, 2012; Longford et al., 2019; Kizhakkekalam & Chakraborty, 2020; Kuba et al., 2021). Diversity within Chlorophyta and Rhodophyta was higher compared to Ochrophyta species, supporting the overall trend observed at the phylum level. Bacterial taxa associated with Ochrophyta species were less diverse based on Simpson’s, Shannon, and Faith’s phylogenetic diversity indices compared to species of Chlorophyta and Rhodophyta, potentially due to secondary metabolite production by Ochrophyta selecting for microbiota with antimicrobial capabilities (Ismail et al., 2016). The Ochrophyta-associated microbiota were also less relatively abundant than in the surrounding seawater, similar to previous findings with *Laminaria saccharina* (Ochrophyta) sampled from the Baltic and North Seas (Ismail et al., 2016). Ochrophyta-associated microbial communities were more similar to one another than those associated with the other two phyla, suggesting that Ochrophyta may be more selective in their microbial counterparts and further supporting the variation of microbial counterparts among macroalgal hosts (Saha & Weinberger, 2019). Ochrophyta were infrequently encountered in the subtidal zone and were only collected in the upper mesophotic zone in this study. Further study of the same species collected herein across depths is warranted to better understand these trends given that patterns in macroalgal diversity have been shown to correspond with solar irradiance and depths associated with seasonal thermoclines, influencing the microbial species and phyla that are observed at specific depth zones (Lesser, Slattery & Leichter, 2009; Wiencke & Bischof, 2012).

A similar study was completed at ‘Ewa Beach, HI, an intertidal bench that is anthropogenically altered (Kuba et al., 2021). The taxonomic composition of macroalgal-associated bacteria at Manawai was similar to that found previously at ‘Ewa Beach (Kuba et al., 2021), with Actinobacteriota, Bacteroidota, Myxococcota, Alphaproteobacteria, Verrucomicrobiota and Cyanobacteria also being identified at Manawai. The macroalgal-microbial communities at ‘Ewa Beach however, included Bdellovibrionota (Kuba et al., 2021), which was not found to be associated with the macroalgae collected at Manawai. Bacteriodota, Alphaproteobacteria, and Verrucomicrobiota were less relatively abundant compared to ‘Ewa Beach (Kuba et al., 2021). Differences in composition are important to note as small- (*e.g*., macroalgal individual) and large-scale (*e.g*., Manawai) environments may be influencing microbial community structure. Overall, the macroalgal microbial communities from ‘Ewa Beach and Manawai showed similar average diversity for both Simpson’s (‘Ewa Beach = 0.79; Manawai = 0.80) and Shannon (‘Ewa Beach = 4.27; Manawai = 4.20) diversity indices (Kuba et al., 2021). Similar microbial diversity across these sites suggests that local influences may not have major impacts on larger-scale biodiversity patterns. The most relatively abundant bacterial taxa associated with all algae at Manawai (Hyphomonas, Rhodobacteraceaes, and Stenotrophomonas) are metabolically diverse as denitrifiers, diazotrophs, or can utilize nitrate in the absence of oxygen (Weiner, Devine & Powell, 1985; Lesser, Slattery & Leichter, 2009), and thus may be related to nitrogen cycling. The presence of these microbiota in the nearly pristine ecosystem of the PMNM however, may lend support for their natural associations with macroalgae, rather than previous findings as nitrogen-fixing bacteria that proliferate in response to increased bioavailable nitrogen from anthropogenic sources (Lapointe & Bedford, 2011).

With the decline of shallow-water reef ecosystems, research investigating MCEs as potential refugia to support recovery of impacted shallow reefs has increased (Bongaerts et al., 2010). MCEs were initially proposed as a refuge/refugia for one or multiple species to avoid near-shore disturbances in shallow habitats (Bongaerts et al., 2010). MCEs rely on water clarity and therefore typically occur in offshore environments where the water is generally less turbid (Bak, Nieuwland & Meesters, 2005; Spalding et al., 2012). As depth increases, water motion from wave exposure decreases and water temperatures become more consistent, especially in thermally stable months (February to May) (Pyle & Kosaki, 2016). The NWHI are exposed to winter swells and multiple extreme wave events reaching maximum depths of approximately 20 m (Grigg et al., 2008). In turn, shallow community structure may be more homogenous due to high levels of disturbance. *M. setchellianum* was associated with significantly different microbial communities across six sites, which also varied in depth zone due to the opportunistic nature of our sampling, suggesting limited connectivity among these sites or between shallow and mesophotic depths. The separation of microbial-macroalgal relationships based on depth zones would support the unique nature of mesophotic systems rather than their role as shallow water refugia based on macroalgal-associated microbial communities at Manawai.

At 22.5 m depth, *M. setchellianum* had more individual variation in microbial communities compared to *H. velasquezii*, which may be attributed to differences in their morphological complexity (Morrissey et al., 2019). Host morphology influences associated microbial communities, where microbial richness increases with morphological complexity (Lemay et al., 2021; Kuba et al., 2021). *M. setchellianum* has an uncalcified, net-like thallus structure that forms dense beds, offering a complex surface area that may capture organic matter and provide a more favorable habitat for microbial colonizers. Photosynthetic productivity of *M. setchellianum* also decreases considerably by 100 m depth, suggesting a slower growth rate and longer-lived thalli for microbial community formation (Runcie, Gurgel & Mcdermid, 2008). The overall morphological structure of *Halimeda* compared to the morphological complexity of *M. setchellianum* also may contribute to the microbial community dynamics between these two macroalgal genera because of the surface area and type of surface provided by each. However, morphology as a driver of microbial community could not be directly tested due to the variation of algal species sampled across different sites and depths.

In the current study, cryptic fish species were observed using *M. setchellianum* beds for habitat, which was not observed in the scattered individuals of *Halimeda* spp. *Halimeda* is a calcareous, coenocytic genus with a single, multinucleate cell (Drew & Abel, 1988; Vroom & Smith, 2001), and the smooth calcareous surface of *Halimeda* spp. individuals offers a physical defense with less surface area for colonization. Growth of *Halimeda* occurs apically *via*, new segments, with calcification of these new segments occurring 24–48 h after segment formation (Hillis-Colinvaux, 1980). *Halimeda* individuals therefore have older, perennial bases typically characterized by epiphytic growth, whereas the younger segments are less epiphytized due to less settlement time (Hillis-Colinvaux, 1980). *Halimeda* microbiome collections herein were derived from the newer apical segments, potentially limiting the overall microbial communities of these species. The younger age of apical *Halimeda* segments may limit the establishment of stable microbial relationships. Even though both species of *Halimeda* have the same growth patterns, comparisons revealed differences at the host species-level. Microbial studies across the entire thallus are needed to determine the effect of thalli age on microbial community structure. The microbial communities associated with *H. discoidea* in the intertidal zone at ‘Ewa Beach in the Main Hawaiian Islands hosted a higher abundance and more diverse bacteria than those at Manawai. The average Simpson’s index for *H. discoidea* collected at ‘Ewa Beach was 0.973 (Kuba et al., 2021), which was similar to the average of those collected at Manawai (0.961).

*Chondria tumulosa* is a mat-forming macroalga exhibiting invasive-like traits by over-growing benthic organisms on the reef at Manawai (Sherwood et al., 2020). *Chondria tumulosa* was the only species identified as invasive-like and cryptogenic in this study, whereas all other species were characterized as native at Manawai. Microbiota associated with this cryptogenic alga clustered together to the exclusion of other native Rhodophyta when accounting for collection location. Although this may not be fully attributed to the invasive-like nature of this species, this clustering is important to note for overall microbial biodiversity changes with the proliferation of *C. tumulosa. Chondria tumulosa* also had a lower microbial diversity when compared to other Rhodophyta species which may be indicative of the species’ higher selectivity, as compared to the other red algal species analyzed (Saha & Weinberger, 2019). Bonthond et al. (2020) reported that the microbiota associated with the invasive red alga *Gracilaria vermicullophyla* were highly influenced by the ambient environment. The observed differences in associated microbiota in the current study may also be associated with possible co-introduced microbes if *C. tumulosa* is truly non-native, which could promote its invasiveness (Bonthond et al., 2020). If this cryptogenic alga continues to increase in abundance, then overall species and functional diversity at Manawai is anticipated to diminish. It is important to note that the collection of *C. tumulosa* in this study was not extensive (*n* = 2 sites), despite its proliferation across the atoll. This opportunistic sampling makes it difficult to attribute microbial differences only to the cryptogenic status of the species at Manawai. Additional collections of *C. tumulosa* and related species like *L. galtsoffii* from the same sites are needed to better understand if the cryptogenic status of this alga is influencing the associated microbiota of other macroalgae, thus altering the reef microbiota in impacted areas.

## Conclusions

Examining the entire host-associated microbiota at multiple sites and depths around a protected and isolated atoll provides further insight into the characterization of macroalgal-associated microbial species. Microbial communities at Manawai vary across depth, site, and significantly differed for *C. tumulosa*; however additional studies are required to identify if the cryptogenic status of this alga is a driving factor for these differences. This study investigated the macroalgal-microbial relationships at a remote atoll in the Northwestern Hawaiian Islands and identified biological diversity within subtidal and mesophotic depths. Future studies should focus on the same macroalgal species across different depths to further understand the continuity between subtidal and mesophotic ecosystems. More comparisons across collection depths are needed but are limited because of the number of currently described macroalgal species at upper mesophotic depths (55, 58, and 75 m) compared to subtidal depths (13, 22.5, and 27 m) (Spalding et al., 2019). Direct comparisons of the same macroalgal species between the MHI and PMNM would elucidate the core and variable microbiome of the species that are most abundant in these regions. Comparisons of macroalgal communities from the same macroalgal species across the Hawaiian Archipelago would also provide spatial resolution of these trends. Further investigations across morphological complexity of the macroalgal host would increase the understanding of microbial-macroalgal relationships. This study provides a foundation for macroalgal-microbial studies at Manawai. Although our analyses only begin to characterize these relationships, future studies will further explore macroalgal associated microbiota. Overall, this study highlights the microbial disconnect between shallow and mesophotic coral reefs and the need for further studies of intact ecosystems such as the PMNM.

## Supplemental Information

10.7717/peerj.16114/supp-1 Supplemental Information 1 Rarefaction curves of bacterial partial SSU ribosomal RNA gene sequences for macroalgal samples and seawater controls. phyla are indicated by colors with each shade indicative of a separate species: Chlorophyta (green), Ochrophyta (brown), Rhodophyta (red). Background seawater samples (blue) are also included. Sampling depth associated with the fewest number of sequences are indicated by the black line.

10.7717/peerj.16114/supp-2 Supplemental Information 2 Boxplots of Shannon-Weiner diversity (Shannon) and Faith’s Phylogenetic Diversity (PD) indices. Asterisks indicate significant differences based on Kruskal Wallis pairwise comparisons. ANOVA ran on Shannon (*p* = <0.001) and PD (*p* = <0.001) were both statistically significant.

10.7717/peerj.16114/supp-3 Supplemental Information 3 Non-metric multidimensional scaling plot generated using Bray-Curtis dissimilarities for microbial communities associated with all macroalgal species collected. Species are color labeled by their associated phylum (*n* = 77) with each shape depicting the depth zone that each sample was collected at (shallow subtidal, subtidal, and upper mesophotic).

10.7717/peerj.16114/supp-4 Supplemental Information 4 Non-metric multidimensional scaling plot generated using Bray-Curtis dissimilarities for microbial communities associated with macroalgal species collected at 2 m depth. Species are color labeled. *Chondria tumulosa* (*n* = 3) is noted as a cryptogenic species at this atoll, whereas *Laurencia galtsoffii* (*n* = 3) is native.

10.7717/peerj.16114/supp-5 Supplemental Information 5 Macroalgal sample identification and voucher verification. Molecular identification was completed using either the *tuf*A. mitochondrial *COI*, or *rbcL* gene.

10.7717/peerj.16114/supp-6 Supplemental Information 6 Light and temperature characteristics at collection depths. *K_0_* was calculated using the average irradiance profiles of six locations over six days according to Beer’s Law: y = 1270.2^−0.118x^. The average *K_0_* from all irradiance profiles was used in the following calculations. The average *K_0_* from all irradiance profiles was used in the following calculations. The % Subsurface Irradiance was calculated from irradiance extrapolated from *K_0_* at 0.01 m.

10.7717/peerj.16114/supp-7 Supplemental Information 7 Observed amplicon sequence variants (ASVs) of bacterial taxa diversity indices of the microbial communities associated with macroalgal species. Diversity indices include Shannon-Weiner, Simpson, Inverse Simpson, and Faith’s Phylogenetic Diversity Index.

10.7717/peerj.16114/supp-8 Supplemental Information 8 P-values associated with microbial diversity indices between macroalgal phylum and seawater controls. Highlighted rows are those relationships that have a significant difference in macroalgal microbial diversity. All relationships with seawater are significantly different except the Faith’s Phylogenetic Diversity index between Ochrophyta and Seawater.

## Additional Information and Declarations

### Field Study Permissions

The following information was supplied relating to field study approvals (*i.e*., approving body and any reference numbers): Field collections were approved by the Papahānaumokuākea Marine National Monument, PMNM-2018-029.

### DNA Deposition

The following information was supplied regarding the deposition of DNA sequences: The sequence data are available at NCBI Sequence Read Archive (SRA) BioProject: PRJNA833318.

### Data Availability

The following information was supplied regarding data availability: The data is available at Zenodo: fullertonhe. (2023). fullertonhe/NWHI_MacroalgaeMicrobiomes: NWHI_MacroalgaeMicrobiome (microbiome). Zenodo. https://doi.org/10.5281/zenodo.7975190.

## References

[ref-1] Abbott IA (1999). Marine red algae of the Hawaiian Islands.

[ref-2] Abbott IA, Huisman JM (2004). Marine green and brown algae of the Hawaiian Islands.

[ref-3] Abbott IA, McDermid KJ (2002). On two species of *Kallymenia* (Rhodophyta: Gigartinales: Kallymeniaceae) from the Hawaiian Islands, Central Pacific. Pacific Science.

[ref-4] Aires T, Serrão EA, Kendrick G, Duarte CM, Arnaud-Haond S (2013). Invasion is a community affair: clandestine followers in the bacterial community associated to green algae, *Caulerpa racemosa*, track the invasion source. PLOS ONE.

[ref-5] Armstrong E, Yan L, Boyd KG, Wright PC, Burgess JG (2001). The symbiotic role of marine microbes on living surfaces. Hydrobiologia.

[ref-6] Bak R, Nieuwland G, Meesters E (2005). Coral reef crisis in deep and shallow reefs: 30 years of constancy and change in reefs of Curacao and Bonaire. Coral Reefs.

[ref-7] Bongaerts P, Ridgway T, Sampayo EM, Hoegh-Guldberg O (2010). Assessing the “deep reef refugia” hypothesis: focus on Caribbean reefs. Coral Reefs.

[ref-8] Bonthond G, Bayer T, Krueger-Hadfield SA, Barboza FR, Nakaoka M, Valero M, Wang G, Künzel S, Weinberger F (2020). How do microbiota associated with an invasive seaweed vary across scales?. Molecular Ecology.

[ref-9] Bonthond G, Bayer T, Krueger-Hadfield SA, Stärck N, Wang G, Nakaoka M, Künzel S, Weinberger F (2021). The role of host promiscuity in the invasion process of a seaweed holobiont. The ISME Journal.

[ref-10] Boström KH, Simu K, Hagström Å, Riemann L (2004). Optimization of DNA extraction for quantitative marine bacterioplankton community analysis. Limnology and Oceanography: Methods.

[ref-11] Burke C, Steinberg P, Rusch D, Kjelleberg S, Thomas T (2011a). Bacterial community assembly based on functional genes rather than species. Proceedings of the National Academy of Sciences of the United States of America.

[ref-12] Burke C, Thomas T, Lewis M, Steinberg P, Kjelleberg S (2011b). Composition, uniqueness and variability of the epiphytic bacterial community of the green alga *Ulva australis*. International Society for Microbial Ecology Journal.

[ref-13] Buschmann AH (1990). Intertidal macroalgae as refuge and food for amphipoda in Central Chile. Aquatic Botany.

[ref-14] Busetti A, Maggs CA, Gilmore BF (2017). Marine macroalgae and their associated microbiomes as a source of antimicrobial chemical diversity. European Journal of Phycology.

[ref-15] Callahan BBJ, McMurdie PJP, Rosen MJM, Han AW, Johnson AAJA, Holmes SP (2016). DADA2: high-resolution sample inference from Illumina amplicon data. Nature Methods.

[ref-16] Clarke KR, Gorley RN (2001). PRIMER v5: user manual/tutorial.

[ref-17] Dobretsov S, Dahms H, Harder T (2006). Allelochemical defense against epibiosis in the macroalga Caulerpa racemosa var. turbinata. Marine Ecology Progress Series.

[ref-18] Drew EA, Abel KM (1988). Studies on Halimeda. Coral Reefs.

[ref-19] Edgar RC (2004). MUSCLE: a multiple sequence alignment method with reduced time and space complexity. BMC Bioinformatics.

[ref-20] Egan S, Thomas T, Kjelleberg S (2008). Unlocking the diversity and biotechnological potential of marine surface associated microbial communities. Current Opinion in Microbiology.

[ref-21] Fraschetti S, Terlizzi A, Bevilacqua S, Boero F (2006). The distribution of hydroids (Cnidaria, Hydrozoa) from micro- to macro-scale: spatial patterns on habitat-forming algae. Journal of Experimental Marine Biology and Ecology.

[ref-22] Friedlander A, Aeby G, Brainard R, Clark A, DeMartini E, Godwin S, Kenyon J, Kosaki R, Maragos J, Vroom P (2005). The state of coral reef ecosystems of the Northwestern Hawaiian Islands. The State of Coral Reef Ecosystems of the United States and Pacific Freely Associated States.

[ref-23] Fukunaga A (2008). Invertebrate community associated with the macroalga Halimeda kanaloana Meadow in Maui, Hawaii. International Review of Hydrobiology.

[ref-24] Gavio B, Fredericq S (2002). Grateloupia turuturu (Halymeniaceae, Rhodophyta) is the correct name of the non-native species in the Atlantic known as Grateloupia doryphora. European Journal of Phycology.

[ref-25] Goecke F, Labes A, Wiese J, Imhoff J (2010). Chemical interactions between marine macroalgae and bacteria. Marine Ecology Progress Series.

[ref-26] Grigg RW, Polovina J, Friedlander AM, Rohmann SO (2008). Biology of coral reefs in the Northwestern Hawaiian Islands. Coral Reefs of the USA.

[ref-27] Haas AF, Fairoz MFM, Kelly LW, Nelson CE, Dinsdale EA, Edwards RA, Giles S, Hatay M, Hisakawa N, Knowles B, Lim YW, Maughan H, Pantos O, Roach TNF, Sanchez SE, Silveira CB, Sandin S, Smith JE, Rohwer F (2016). Global microbialization of coral reefs. Nature Microbiology.

[ref-28] Handeler K, Wagele H, Wahrmund U, Rudinger M, Knoop V (2010). Slugs’ last meals: molecular identification of sequestered chloroplasts from different algal origins in Sacoglossa (Opisthobranchia, Gastropoda). Molecular Ecology Resources.

[ref-29] Harney JN, Fletcher CH (2003). A budget of carbonate framework and sediment production, Kailua Bay, Oahu, Hawaii. Journal of Sedimentary Research.

[ref-30] Hay ME (1986). Associational plant defenses and the maintenance of species diversity: turning competitors into accomplices. The American Naturalist.

[ref-31] Haywood MDE, Vance DJ, Loneragan NR (1995). Seagrass and algal beds as nursery habitats for tiger prawns (Penaeus semisulcatus and P. esculentus) in a tropical Australian estuary. Marine Biology.

[ref-32] Hillis-Colinvaux L (1980). Advances in marine biology.

[ref-33] Hinderstein L, Marr J, Martinez F, Dowgiallo M (2010). Mesophotic coral ecosystems: characterization, ecology, and management. Coral Reefs.

[ref-34] Huisman JM, Abbott IA, Smith CM (2007). Hawaiian reef plants.

[ref-35] Ismail A, Ktari L, Ahmed M, Bolhuis H, Boudabbous A, Stal LJ, Cretoiu MS, El Bour M (2016). Antimicrobial activities of bacteria associated with the brown alga Padina pavonica. Frontiers in Microbiology.

[ref-36] Kim MS, Kim SY, Nelson W (2010). *Symphyocladia lithophila* sp. nov. (Rhodomelaceae, Ceramiales), a new Korean red algal species based on morphology and *rbc*L sequences. Botanica Marina.

[ref-37] Kirk J (1994). Light and photosynthesis in aquatic ecosystems.

[ref-38] Kizhakkekalam VK, Chakraborty K (2020). Marine macroalgae-associated heterotrophic Firmicutes and Gamma-proteobacteria: prospective anti-infective agents against multidrug resistant pathogens. Archives of Microbiology.

[ref-39] Klindworth A, Pruesse E, Schweer T, Peplies J, Quast C, Horn M, Glöckner FO (2013). Evaluation of general 16S ribosomal RNA gene PCR primers for classical and next-generation sequencing-based diversity studies. Nucleic Acids Research.

[ref-40] Kuba GM, Spalding HL, Hill-Spanik KM, Fullerton H (2021). Microbiota-macroalgal relationships at a Hawaiian intertidal bench are influenced by macroalgal phyla and associated thallus complexity. mSphere.

[ref-41] Lahti L, Shetty S (2017). Introduction to the microbiome R package. https://microbiome.github.io/tutorials/.

[ref-42] Langston R, Spalding HL (2017). A survey of fishes associated with Hawaiian deep-water Halimeda kanaloana (Bryopsidales: Halimedaceae) and Avrainvillea sp.(Bryopsidales: Udoteaceae). PeerJ.

[ref-43] Lapointe BE, Bedford BJ (2011). Stormwater nutrient inputs favor growth of non-native macroalgae (Rhodophyta) on O’ahu, Hawaiian Islands. Harmful Algae.

[ref-44] Lemay MA, Chen MY, Mazel F, Hind KR, Starko S, Keeling PJ, Martone PT, Parfrey LW (2021). Morphological complexity affects the diversity of marine microbiomes. The ISME Journal.

[ref-45] Lesser MP, Slattery M, Leichter JJ (2009). Ecology of mesophotic coral reefs. Journal of Experimental Marine Biology and Ecology.

[ref-46] Longford SR, Campbell AH, Nielsen S, Case RJ, Kjelleberg S, Steinberg PD (2019). Interactions within the microbiome alter microbial interactions with host chemical defences and affect disease in a marine holobiont. Scientific Reports.

[ref-47] Love MI, Huber W, Anders S (2014). Moderated estimation of fold change and dispersion for RNA-seq data with DESeq2. Genome Biology.

[ref-48] McMurdie PJ, Holmes S (2013). Phyloseq: an R package for reproducible interactive analysis and graphics of microbiome census data. PLOS ONE.

[ref-49] McMurdie PJ, Holmes S (2014). Waste not, want not: why rarefying microbiome data is inadmissible. PLOS Computational Biology.

[ref-50] Morrissey KL, Çavas L, Willems A, De Clerck O (2019). Disentangling the influence of environment, host specificity and thallus differentiation on bacterial communities in siphonous green seaweeds. Frontiers in Microbiology.

[ref-51] Oksanen J, Blanchet FG, Friendly M, Kindt R, Legendre P, Mcglinn D (2016). Package ‘Vegan’—community ecology package, version 2.0–4. https://github.com/vegandevs/vegan/.

[ref-52] Page K, Smith C, Vroom P, Sherwood A (2006). Factors influencing benthic distributional patterns in a near-pristine coral reef ecosystem: Pearl and Hermes Atoll.

[ref-53] Page-Albins KN, Vroom PS, Hoeke R, Albins MA, Smith CM (2012). Patterns in benthic coral reef communities at Pearl and Hermes Atoll along a wave-exposure gradient. Pacific Science.

[ref-54] Parrish FA, Boland RC (2004). Habitat and reef-fish assemblages of banks in the Northwestern Hawaiian Islands. Marine Biology.

[ref-55] Persson F, Svensson R, Nylund GM, Fredriksson NJ, Pavia H, Hermansson M (2011). Ecological role of a seaweed secondary metabolite for a colonizing bacterial community. Biofouling.

[ref-56] Pyle RL, Kosaki RK (2016). Prognathodes basabei, a new species of butterflyfish (Perciformes, Chaetodontidae) from the Hawaiian archipelago. ZooKeys.

[ref-100] R Core Team (2010). R: A language and environment for statistical computing. https://www.R-project.org/.

[ref-57] Runcie JW, Gurgel CFD, Mcdermid KJ (2008). *In situ* photosynthetic rates of tropical marine macroalgae at their lower depth limit. European Journal of Phycology.

[ref-58] Saha M, Ferguson RMW, Dove S, Künzel S, Meichssner R, Neulinger SC, Petersen FO, Weinberger F (2020). Salinity and time can alter epibacterial communities of an invasive seaweed. Frontiers in Microbiology.

[ref-59] Saha M, Weinberger F (2019). Microbial “gardening” by a seaweed holobiont: surface metabolites attract protective and deter pathogenic epibacterial settlement. Journal of Ecology.

[ref-60] Saunders GW (2005). Applying DNA barcoding to red macroalgae: a preliminary appraisal holds promise for future applications. Philosophical Transactions of the Royal Society B: Biological Sciences.

[ref-61] Schiel DR, Foster MS (2006). The population biology of large brown seaweeds: ecological consequences of multiphase life histories in dynamic coastal environments. Annual Review of Ecology, Evolution, and Systematics.

[ref-62] Sherwood AR, Huisman JM, Paiano MO, Williams TM, Kosaki RK, Smith CM, Giuseffi L, Spalding HL (2020). Taxonomic determination of the cryptogenic red alga, Chondria tumulosa sp. nov., (Rhodomelaceae, Rhodophyta) from Papahānaumokuākea Marine National Monument, Hawai‘i, USA: a new species displaying invasive characteristics. PLOS ONE.

[ref-63] Singh RP, Reddy CRK (2014). Seaweed–microbial interactions: key functions of seaweed-associated bacteria. FEMS Microbiology Ecology.

[ref-64] Singh RP, Reddy CRK (2016). Unraveling the functions of the macroalgal microbiome. Frontiers in Microbiology.

[ref-65] Spalding HL, Amado-Filho GM, Bahia RG, Ballantine DL, Fredericq S, Leichter JJ, Nelson WA, Slattery M, Tsuda RT (2019). Macroalgae. Mesophotic Coral Ecosystems.

[ref-66] Spalding HL, Foster MS, Heine JN (2003). Compositional, distribution, and abundance of deep-water (>30m) macroalgae in central California. Journal of Phycology.

[ref-67] Spalding HL, Smith CM, Foster MS, Vroom PS, Hunter CL, Sansone FJ (2012). Ecology of mesophotic macroalgae and Halimeda kanaloana meadows in the main Hawaiian Islands.

[ref-68] Stratil SB, Neulinger SC, Knecht H, Friedrichs AK, Wahl M (2013). Temperature-driven shifts in the epibiotic bacterial community composition of the brown macroalga Fucus vesiculosus. MicrobiologyOpen.

[ref-69] Vroom PS, Page KN, Peyton KA, Kukea-Shultz JK (2005). Spatial heterogeneity of benthic community assemblages with an emphasis on reef algae at French Frigate Shoals, Northwestern Hawai’ian Islands. Coral Reefs.

[ref-70] Vroom P, Smith C (2001). The challenge of siphonous green algae. American Scientist.

[ref-71] Wahl M, Goecke F, Labes A, Dobretsov S, Weinberger F (2012). The second skin: ecological role of epibiotic biofilms on marine organisms. Frontiers in Microbiology.

[ref-72] Weiner RM, Devine RA, Powell DM (1985). Hyphomonas oceanitis sp. nov., Hyphomonas Hirschiana sp. nov. and Hyphomonas jannaschiana sp. nov. International Journal of Systematic Bacteriology.

[ref-73] Wickham H (2016). ggplot2: elegant graphics for data analysis.

[ref-74] Wiencke C, Bischof K (2012). Seaweed biology.

