# Supplementary figures and images for: Characterization of macroalgal-associated microbial communities from shallow to mesophotic depths at Manawai, Papahānaumokuākea Marine National Monument, Hawai‘i
Source: PeerJ. 2023 Oct 3;11:e16114. doi: 10.7717/peerj.16114 (PMC10569167; doi:10.7717/peerj.16114)

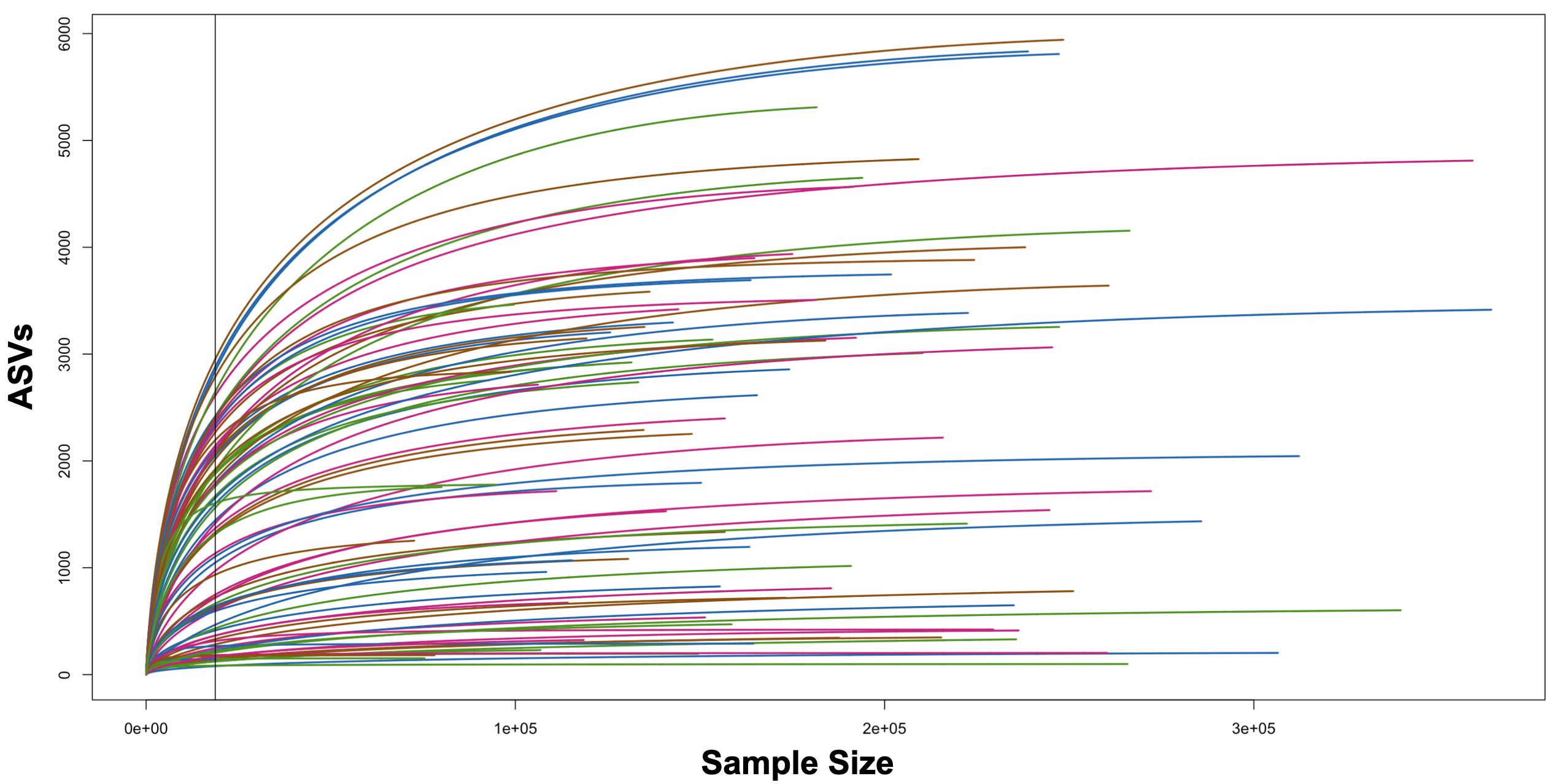

Supplement: Supplemental Information 1 — phyla are indicated by colors with each shade indicative of a separate species: Chlorophyta (green), Ochrophyta (brown), Rhodophyta (red). Background seawater samples (blue) are also included. Sampling depth associated with the fewest number of sequences are indicated by the black line. [file peerj-11-16114-s001.png]

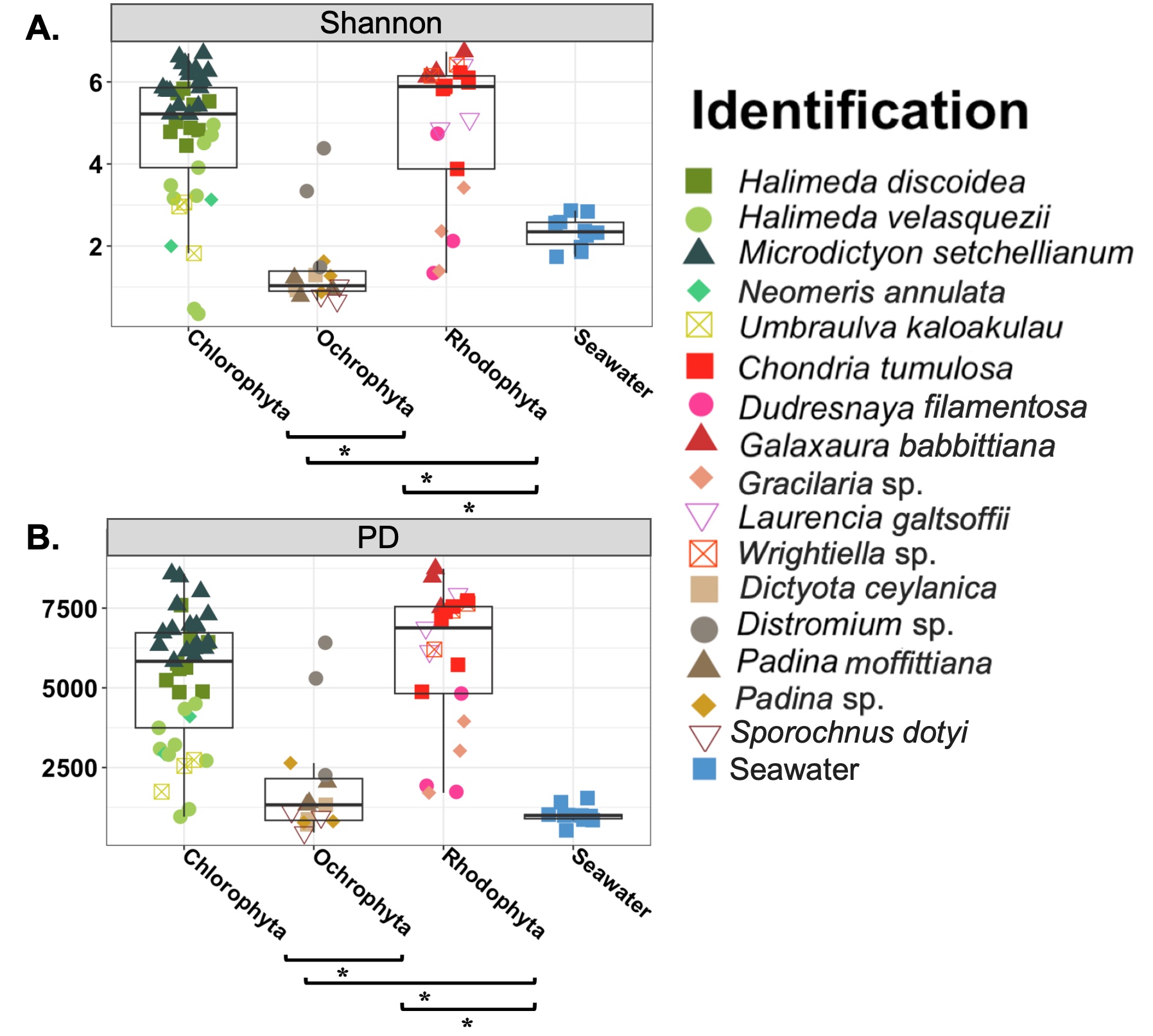

Supplement: Supplemental Information 2 — Asterisks indicate significant differences based on Kruskal Wallis pairwise comparisons. ANOVA ran on Shannon (p = <0.001) and PD (p = <0.001) were both statistically significant. [file peerj-11-16114-s002.jpg]

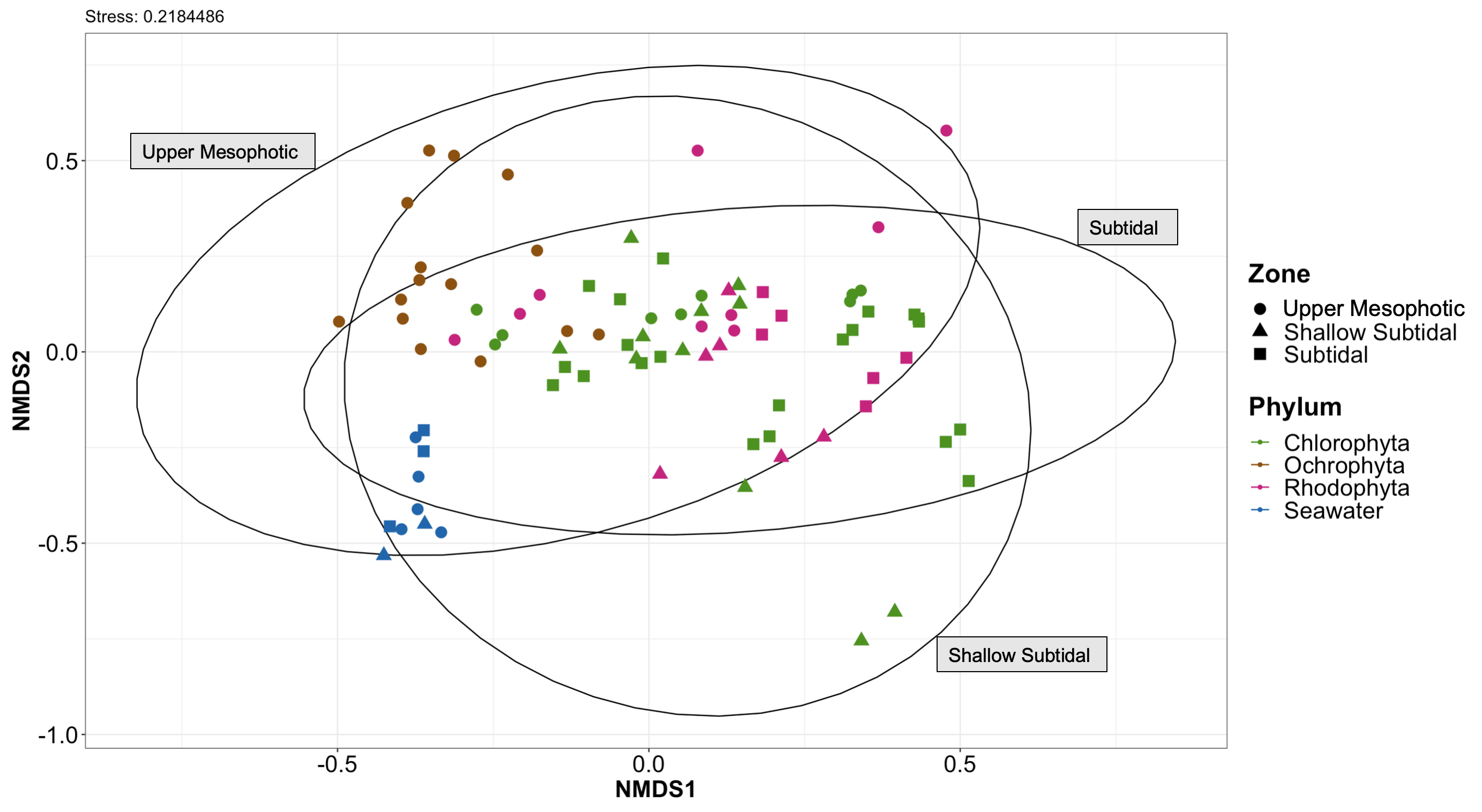

Supplement: Supplemental Information 3 — Species are color labeled by their associated phylum (n = 77) with each shape depicting the depth zone that each sample was collected at (shallow subtidal, subtidal, and upper mesophotic). [file peerj-11-16114-s003.png]

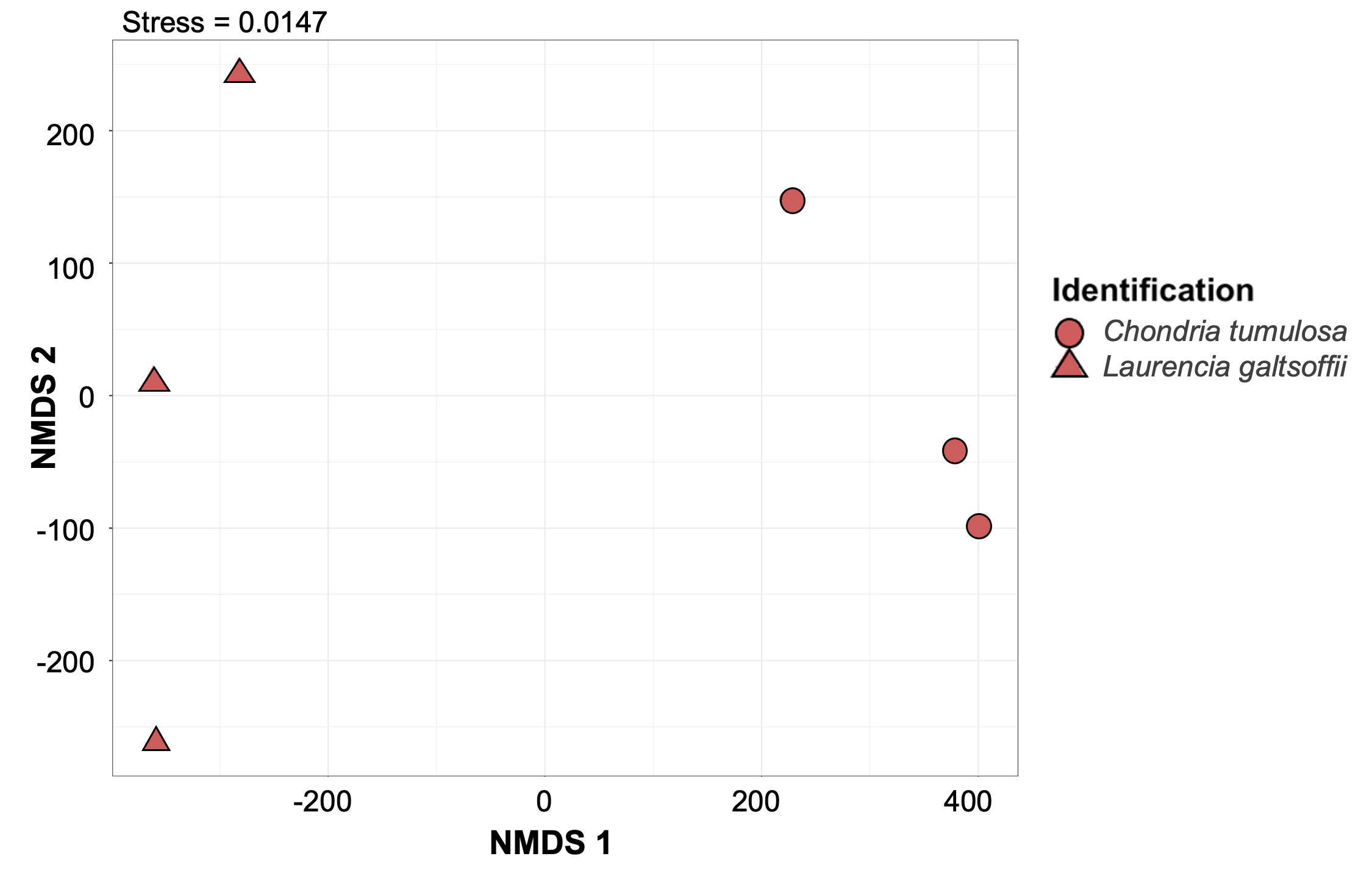

Supplement: Supplemental Information 4 — Species are color labeled. Chondria tumulosa (n = 3) is noted as a cryptogenic species at this atoll, whereas Laurencia galtsoffii (n = 3) is native. [file peerj-11-16114-s004.png]
