# Supplementary material for: Characterization of macroalgal-associated microbial communities from shallow to mesophotic depths at Manawai, Papahānaumokuākea Marine National Monument, Hawai‘i
Source: PeerJ. 2023 Oct 3;11:e16114. doi: 10.7717/peerj.16114 (PMC10569167; doi:10.7717/peerj.16114)
Supplement: Supplemental Information 5 — Molecular identification was completed using either the tufA. mitochondrial COI, or rbcL gene. [file peerj-11-16114-s005.docx]

| **Macroalgal Species** | **Voucher** | **Sample Verification** | **Collection Depth** | **Identification** | **Target Gene** | **GenBank Accession** |
| --- | --- | --- | --- | --- | --- | --- |
| *Halimeda velasquezii* | ARS10534 | NWHI-878 | 13 | Molecular | *tufA* | OR066433 |
| *Galaxaura filamentosa* | ARS10209 | NWHI-904 | 55 | Molecular | *LSU, rbcL* | OR066431, OR066432 |
| *Dudresnaya babbittiana* | ARS09952 | NWHI-909 | 55 | Molecular | *COI, rbcL* | OK448437, OK448460 |
| *Laurencia galtsoffii* | ARS10930 | NWHI-813 | 2 | Molecular | *COI* | OR066437 |
| *Halimeda velasquezii* | ARS10534 | NWHI-878 | 2 | Molecular | *tufA* | OR066433 |
| *Dictyota ceylanica* |  | NWHI-951 | 55 | Morphological | NA | NA |
| *Microdictyon setchellianum* |  | NWHI-989 | 58 | Morphological | NA | NA |
| *Padina moffittiana* |  | NWHI-994 | 58 | Morphological | NA | NA |
| *Sporochnus dotyi* |  | NWHI-984 | 58 | Morphological | NA | NA |
| *Umbraulva kaloakulau* | ARS10921 | NWHI-1049 | 75 | Molecular | *tufA* | OR066435 |
| *Gracilaria* sp. | ARS10929 | NWHI-1032 | 75 | Molecular | *COI* | OR066436 |
| *Halimeda velasquezii* | ARS10539 | NWHI-1071 | 22.5 | Molecular | *tufA* | OR066434 |
| *Halimeda discoidea* |  | NWHI-1072 | 22.5 | Morphological | NA | NA |
| *Microdictyon setchellianum* |  | NWHI-1074 | 22.5 | Morphological | NA | NA |
| *Wrightiella* sp. | ARS10015 | NWHI-1073 | 22.5 | Molecular | *rbcL* | OR066430 |
