# Supplementary material for: Characterization of macroalgal-associated microbial communities from shallow to mesophotic depths at Manawai, Papahānaumokuākea Marine National Monument, Hawai‘i
Source: PeerJ. 2023 Oct 3;11:e16114. doi: 10.7717/peerj.16114 (PMC10569167; doi:10.7717/peerj.16114)
Supplement: Supplemental Information 6 — K0 was calculated using the average irradiance profiles of six locations over six days according to Beer’s Law: y = 1270.2−0.118x. The average K0 from all irradiance profiles was used in the following calculations. The average K0 from all irradiance profiles was used in the following calculations. The % Subsurface Irradiance was calculated from irradiance extrapolated from K0 at 0.01 m. [file peerj-11-16114-s006.docx]

| **Depth Zone** | **Collection Sites** | **Collection Depth (m)** | **Irradiance** | **% Subsurface Irradiance**  **(%SSI)** | | **Temperature**  **(ºC)** | |
| --- | --- | --- | --- | --- | --- | --- | --- |
| **Shallow Subtidal** | H | 1.5 | 1066.00 | | 84.023 | 26-27 |  |
|  | D | 2 | 1003.18 | | 79.071 |  |  |
| **Subtidal** | A | 13 | 273.95 | | 21.593 | 25-27 |  |
|  | B | 22.5 | 89.45 | | 7.050 |  |  |
|  | I | 27 | 52.51 | | 4.139 |  |  |
| **Upper Mesophotic** | G | 75 | 0.18 | | 0.014 | 22 |  |
| **Upper Mesophotic** | C | 55 | 1.93 | | 0.152 | 22-27 |  |
|  | E | 55 | 1.93 | | 0.152 |  |  |
| **Upper Mesophotic** | F | 58 | 1.35 | | 0.107 | 24 |  |
