# Supplementary material for: Characterization of macroalgal-associated microbial communities from shallow to mesophotic depths at Manawai, Papahānaumokuākea Marine National Monument, Hawai‘i
Source: PeerJ. 2023 Oct 3;11:e16114. doi: 10.7717/peerj.16114 (PMC10569167; doi:10.7717/peerj.16114)
Supplement: Supplemental Information 7 — Diversity indices include Shannon-Weiner, Simpson, Inverse Simpson, and Faith’s Phylogenetic Diversity Index. [file peerj-11-16114-s007.docx]

| **Macroalgal Specimen** | **Observed** | **Shannon** | **Simpson** | **Inverse Simpson** | **Faith’s Phylogenetic Diversity (PD)** |
| --- | --- | --- | --- | --- | --- |
| **Dis.sp-55-1** | 535 | 1.022 | 0.337 | 1.508 | 0.913 |
| **Dis.sp-55-2** | 348 | 1.291 | 0.549 | 2.219 | 0.579 |
| **Dis.sp-55-3** | 229 | 0.926 | 0.444 | 1.8 | 0.83 |
| **Du.ba-55-1** | 825 | 2.122 | 0.568 | 2.312 | 0.568 |
| **Du.ba-55-2** | 808 | 1.339 | 0.355 | 1.549 | 0.355 |
| **Du.ba-55-3** | 2253 | 4.737 | 0.965 | 28.69 | 0.965 |
| **Ha.di-1.5-1** | 3136 | 5.729 | 0.984 | 63.226 | 0.984 |
| **Ha.di-1.5-2** | 2857 | 4.783 | 0.939 | 16.515 | 0.939 |
| **Ha.di-1.5-3** | 2398 | 4.825 | 0.963 | 26.775 | 0.963 |
| **Ha.di-22.5-1** | 3254 | 5.442 | 0.96 | 24.772 | 0.96 |
| **Ha.di-22.5-2** | 3009 | 5.03 | 0.96 | 25.125 | 0.96 |
| **Ha.di-22.5-3** | 2615 | 4.447 | 0.917 | 12.099 | 0.917 |
| **Ha.di-27-1** | 4811 | 5.834 | 0.978 | 46.341 | 0.978 |
| **Ha.di-27-2** | 2290 | 4.88 | 0.965 | 28.962 | 0.965 |
| **Ha.di-27-3** | 2737 | 5.526 | 0.982 | 55.923 | 0.982 |
| **Ha.ve-22.5-1** | 1195 | 3.225 | 0.866 | 7.435 | 0.866 |
| **Ha.ve-22.5-2** | 1717 | 3.163 | 0.851 | 6.725 | 0.851 |
| **Ha.ve-22.5-3** | 1335 | 3.478 | 0.871 | 7.771 | 0.871 |
| **Mi.se-1.5-1** | 2899 | 5.994 | 0.99 | 105.201 | 0.99 |
| **Mi.se-1.5-2** | 3203 | 6.179 | 0.991 | 105.542 | 0.991 |
| **Mi.se-1.5-3** | 3419 | 6.323 | 0.993 | 145.482 | 0.993 |
| **Mi.se-27-1** | 3584 | 6.45 | 0.994 | 174.036 | 0.994 |
| **Mi.se-27-2** | 2756 | 6.031 | 0.99 | 102.275 | 0.99 |
| **Mi.se-27-3** | 3296 | 6.293 | 0.993 | 146.295 | 0.993 |
| **Mi.se-13-1** | 2714 | 5.425 | 0.968 | 31.044 | 0.968 |
| **Mi.se-13-2** | 3147 | 5.774 | 0.979 | 48.358 | 0.979 |
| **Mi.se-13-3** | 2922 | 5.855 | 0.988 | 85.574 | 0.988 |
| **Ne.an-1.5.1** | 781 | 0.944 | 0.241 | 1.317 | 0.796 |
| **Ne.an-1.5.2** | 1017 | 1.225 | 0.308 | 1.446 | 0.525 |
| **Pa.mo-55-1** | 649 | 0.775 | 0.206 | 1.26 | 0.241 |
| **Pa.mo-55-2** | 1540 | 1.626 | 0.45 | 1.818 | 0.308 |
| **Pa.mo-55-3** | 345 | 1.273 | 0.546 | 2.203 | 0.206 |
| **Pa.sp-58-1** | 330 | 0.874 | 0.286 | 1.4 | 0.45 |
| **Pa.sp-58-2** | 961 | 3.059 | 0.795 | 4.87 | 0.546 |
| **Pa.sp-58-3** | 673 | 1.823 | 0.646 | 2.823 | 0.286 |
| **Um.ka-75-1** | 1083 | 2.965 | 0.759 | 4.146 | 0.795 |
| **Um.ka-75-2** | 4155 | 4.382 | 0.913 | 11.545 | 0.646 |
| **Um.ka-75-3** | 1435 | 1.484 | 0.579 | 2.377 | 0.759 |
| **Di.ce-55-1** | 3063 | 3.339 | 0.83 | 5.866 | 0.337 |
| **Di.ce-55-2** | 5942 | 6.738 | 0.994 | 169.09 | 0.549 |
| **Di.ce-55-3** | 4650 | 6.119 | 0.989 | 90.533 | 0.444 |
| **Ga.fi-55-1** | 5833 | 6.252 | 0.985 | 67.464 | 0.994 |
| **Ga.fi-55-2** | 2218 | 3.421 | 0.881 | 8.405 | 0.989 |
| **Ga.fi-55-3** | 719 | 1.389 | 0.401 | 1.668 | 0.985 |
| **Gr.sp-75-1** | 1413 | 2.362 | 0.646 | 2.821 | 0.881 |
| **Gr.sp-75-2** | 3385 | 5.092 | 0.924 | 13.112 | 0.401 |
| **Gr.sp-75-3** | 4565 | 6.41 | 0.989 | 90.784 | 0.646 |
| **La.ga-2-1** | 4000 | 4.863 | 0.907 | 10.767 | 0.924 |
| **La.ga-2-2** | 3463 | 6.42 | 0.993 | 145.064 | 0.989 |
| **La.ga-2-3** | 3692 | 6.147 | 0.989 | 87.294 | 0.907 |
| **Wr.sp-22.5-1** | 3153 | 5.887 | 0.988 | 80.355 | 0.993 |
| **Wr.sp-22.5-2** | 3641 | 5.219 | 0.952 | 21.036 | 0.989 |
| **Wr.sp-22.5-3** | 3254 | 5.414 | 0.97 | 33.054 | 0.988 |
| **Mi.se-22.5-1** | 3415 | 5.209 | 0.967 | 30.688 | 0.952 |
| **Mi.se-22.5-2** | 3937 | 5.781 | 0.981 | 51.66 | 0.97 |
| **Mi.se-22.5-3** | 3126 | 5.86 | 0.985 | 67.5 | 0.967 |
| **Mi.se-58-1** | 5310 | 6.45 | 0.99 | 102.62 | 0.981 |
| **Mi.se-58-2** | 5809 | 6.612 | 0.994 | 156.899 | 0.985 |
| **Mi.se-58-3** | 3898 | 6.261 | 0.992 | 120.466 | 0.99 |
| **Mi.se-55-1** | 4825 | 6.695 | 0.995 | 207.51 | 0.994 |
| **Mi.se-55-2** | 602 | 1.031 | 0.41 | 1.695 | 0.992 |
| **Mi.se-55-3** | 203 | 0.785 | 0.401 | 1.669 | 0.995 |
| **Sp.do-58-1** | 413 | 0.662 | 0.192 | 1.238 | 0.41 |
| **Sp.do-58-2** | 190 | 1.857 | 0.627 | 2.678 | 0.401 |
| **Sp.do-58-3** | 195 | 2.582 | 0.774 | 4.416 | 0.192 |
| **Wa-1.5-10** | 290 | 2.366 | 0.729 | 3.686 | 0.627 |
| **Wa-2-7** | 203 | 1.737 | 0.595 | 2.47 | 0.774 |
| **Wa-13-5** | 182 | 2.56 | 0.753 | 4.05 | 0.729 |
| **Wa-22.5-12** | 153 | 1.974 | 0.664 | 2.972 | 0.595 |
| **Wa-27-4** | 145 | 2.863 | 0.795 | 4.867 | 0.753 |
| **Wa-55-6** | 422 | 2.84 | 0.875 | 8.002 | 0.664 |
| **Wa-55-8** | 185 | 2.325 | 0.774 | 4.432 | 0.795 |
| **Wa-58-9** | 99 | 2.256 | 0.791 | 4.777 | 0.875 |
| **Wa-75-11** | 1070 | 3.91 | 0.95 | 19.856 | 0.774 |
| **Wa-55-1** | 1717 | 4.956 | 0.978 | 44.484 | 0.791 |
| **Ha.ve-13-1** | 1253 | 4.509 | 0.951 | 20.379 | 0.95 |
| **Ha.ve-13-2** | 471 | 0.465 | 0.106 | 1.118 | 0.978 |
| **Ha.ve-13-3** | 2045 | 4.709 | 0.965 | 28.192 | 0.951 |
| **Ha.ve-2-1** | 324 | 0.345 | 0.078 | 1.085 | 0.106 |
| **Ha.ve-2-2** | 2834 | 6.103 | 0.982 | 55.524 | 0.965 |
| **Ha.ve-2-3** | 1778 | 5.899 | 0.984 | 64.119 | 0.078 |
| **Ch.tu-13-1** | 3745 | 6.23 | 0.987 | 78.456 | 0.982 |
| **Ch.tu-13-2** | 3507 | 5.99 | 0.985 | 67.005 | 0.984 |
| **Ch.tu-13-3** | 3881 | 5.827 | 0.977 | 43.805 | 0.987 |
| **Ch.tu-2-1** | 3507 | 5.99 | 0.985 | 67.005 | 0.985 |
| **Ch.tu-2-2** | 3881 | 5.827 | 0.977 | 43.805 | 0.977 |
| **Ch.tu-2-3** | 1755 | 3.878 | 0.842 | 6.32 | 0.842 |
