# Supplementary material for: Characterization of macroalgal-associated microbial communities from shallow to mesophotic depths at Manawai, Papahānaumokuākea Marine National Monument, Hawai‘i
Source: PeerJ. 2023 Oct 3;11:e16114. doi: 10.7717/peerj.16114 (PMC10569167; doi:10.7717/peerj.16114)
Supplement: Supplemental Information 8 — Highlighted rows are those relationships that have a significant difference in macroalgal microbial diversity. All relationships with seawater are significantly different except the Faith’s Phylogenetic Diversity index between Ochrophyta and Seawater. [file peerj-11-16114-s008.docx]

| **Diversity Index** | **Relationship** | **P-value** | **Full Model P-value** |
| --- | --- | --- | --- |
| **Observed ASVs** | Chlorophyta: Ochrophyta | 0.0001 | <0.0001 |
|  | Chlorophyta: Rhodophyta | 0.2300 |  |
|  | Chlorophyta: Seawater | <0.0001 |  |
|  | Rhodophyta: Ochrophyta | <0.0001 |  |
|  | Rhodophyta: Seawater | <0.0001 |  |
|  | Ochrophyta: Seawater | 0.0003 |  |
| **Shannon Index** | Chlorophyta: Ochrophyta | <0.0001 | <0.0001 |
|  | Chlorophyta: Rhodophyta | 0.4400 |  |
|  | Chlorophyta: Seawater | <0.0001 |  |
|  | Rhodophyta: Ochrophyta | <0.0001 |  |
|  | Rhodophyta: Seawater | 0.0013 |  |
|  | Ochrophyta: Seawater | 0.0015 |  |
| **Simpson Index** | Chlorophyta: Ochrophyta | <0.0001 | <0.0001 |
|  | Chlorophyta: Rhodophyta | 0.9900 |  |
|  | Chlorophyta: Seawater | <0.0001 |  |
|  | Rhodophyta: Ochrophyta | <0.0001 |  |
|  | Rhodophyta: Seawater | 0.0042 |  |
|  | Ochrophyta: Seawater | 0.0012 |  |
| **Faith’s Phylogenetic Diversity** | Chlorophyta: Ochrophyta | <0.0001 | <0.0001 |
|  | Chlorophyta: Rhodophyta | 0.1500 |  |
|  | Chlorophyta: Seawater | <0.0001 |  |
|  | Rhodophyta: Ochrophyta | <0.0001 |  |
|  | Rhodophyta: Seawater | <0.0001 |  |
|  | Ochrophyta: Seawater | 0.3700 |  |
